# RNF219 regulates CCR4-NOT function in mRNA translation and deadenylation

**DOI:** 10.1038/s41598-022-13309-8

**Published:** 2022-06-03

**Authors:** Aude Guénolé, Fabien Velilla, Aymeric Chartier, April Rich, Anne-Ruxandra Carvunis, Claude Sardet, Martine Simonelig, Bijan Sobhian

**Affiliations:** 1 Institut de Recherche en Cancérologie de Montpellier (IRCM), INSERM, Université de Montpellier, Institut Régional du Cancer de Montpellier (ICM), 34298 Montpellier, France; 2 Institut de Génétique Humaine, CNRS, Université de Montpellier, 34396 Montpellier, France; 3 Department of Computational and Systems Biology, Pittsburgh Center for Evolutionary Biology and Medicine, School of Medicine, University of Pittsburgh, Pittsburgh, PA 15213 USA

**Keywords:** Translation, Proteomic analysis, RNA, Multienzyme complexes, Gene regulation

## Abstract

Post-transcriptional regulatory mechanisms play a role in many biological contexts through the control of mRNA degradation, translation and localization. Here, we show that the RING finger protein RNF219 co-purifies with the CCR4-NOT complex, the major mRNA deadenylase in eukaryotes, which mediates translational repression in both a deadenylase activity-dependent and -independent manner. Strikingly, RNF219 both inhibits the deadenylase activity of CCR4-NOT and enhances its capacity to repress translation of a target mRNA. We propose that the interaction of RNF219 with the CCR4-NOT complex directs the translational repressive activity of CCR4-NOT to a deadenylation-independent mechanism.

## Introduction

The regulation of gene expression is composed of transcriptional and post-transcriptional events. In eukaryotes, mRNA stability and translation are intimately linked to the 5′ end cap structure and the 3′ end polyadenosine (poly(A)) tail of the mRNA^1^. Eukaryotic mRNA decay is generally initiated by shortening of the poly(A) tail. Then, removal of the 5′ cap structure (decapping) is followed by 5′ to 3′ exonucleolytic degradation or alternatively mRNA is digested from the 3′ end (reviewed in^2,3^). Besides its function in mRNA decay, deadenylation also contributes to translation silencing^1^. In some cases, deadenylated transcripts can be stable but untranslatable until cytoplasmic polyadenylation reactivates translation^4,5^. Moreover, long poly(A) tails induce translation in oocytes and early embryos^6–8^ while in somatic cells this correlation between translation potential and poly(A) tail length is lost^9^. Interestingly, short poly(A) tails have been linked to abundant and highly translated mRNAs^10^.

One of the major components involved in deadenylation is the evolutionary conserved CCR4-NOT complex. The structure–function of most CCR4-NOT subunits has been well characterized in yeast, *Drosophila* and humans^11–16^. These reports showed that CNOT1, the largest CCR4-NOT core subunit, is required to maintain the integrity of the complex. It docks the CNOT2 and CNOT3 subunits at its C-terminus, the deadenylase subunits, CNOT7 and CNOT8, at its central MIF4G domain, and CNOT11/CNOT10 at its N-terminus^12,13,15^. The deadenylase activity of the complex is carried out by the DEDD domain containing exonucleases CNOT7 (CAF1A) and CNOT8 (CAF1B), and the endonuclease-exonuclease-phosphatase (EEP) CNOT6 (CCR4a) and CNOT6L (CCR4b). Only one CAF1 and one CCR4 are found per CCR4-NOT complex^16^.

CCR4-NOT plays a role in global mRNA degradation^3,17^. Yet, it has been more specifically described for targeted mRNA decay such as nonsense-mediated mRNA decay (NMD) where CCR4-NOT is recruited by RNA binding proteins (RBPs) or miRNAs to specific 3′ untranslated regions (UTRs) in mRNAs under certain physiological conditions^18–23^, and during developmental processes^24,25^.

Besides its role in deadenylation-dependent mRNA decay, CCR4-NOT can also regulate translation in a deadenylation-independent manner. In this pathway, the translation repressors DDX6 (*p54/RCK*) (yeast ortholog: Dhh1) and EIF4ENIF1 (4E-T) bind to the CCR4-NOT scaffold subunit CNOT1^26,27^. These proteins can activate the decapping machinery, which leads to translation inhibition, mRNA storage and/or mRNA degradation^28–31^.

Here, we identify the yet incompletely characterized C3HC4 RING domain dependent E3 Ubiquitin ligase RNF219, as a stable interactor of the CCR4-NOT complex. We further show that RNF219 inhibits the translation and stabilizes the poly(A) tail of a reporter mRNA. During the course of our study two groups independently reported RNF219 association with human CCR4-NOT, negatively affecting its enzymatic function^32,33^. Importantly, loss of the interaction between RNF219 and the CCR4-NOT complex abolishes these two activities. Hence, we propose that RNF219 may act as a molecular switch that enables CCR4-NOT to change function from deadenylation-dependent to deadenylation-independent translational repression.

## Materials and methods

### Cell culture

HEK293T, HeLa, and U2OS cells were grown in DMEM (Life Technologies) containing 10% FBS (Sigma-Aldrich) and 1% penicillin/streptomycin (Life Technologies). HEK293T and HeLa RNF219 CRISPR KO (HEK293T SG1-C and HeLa SG1-C) construct and cell lines were generated according to Ran et al.^34^. The sequences used to generate the guide RNA are sgRNA_1F (CACCGCTATGCTAAGCCATACGGTC), sgRNA_1R (AAACGACCGTATGGCTTAGCATAG).

### Antibodies and reagents

Antibodies were obtained from Santa Cruz Biotechnology (Tubulin sc-5286, p27 Antibody (F-8) sc-1641), Bethyl Laboratories (CNOT3 A302-156A, CNOT2 A302-562A, RNF219 A302-540A (RNF219-C), RPL7a A300-749A), Proteintech (CNOT1 14276-1-AP), Covance (anti-HA.11, MMS-101P) and Abcam (GAPDH ab9485). FLAG-M2 agarose (F2426 or A2220-5ML) and FLAG peptide (F3290-4MG) were purchased from Sigma-Aldrich. RNF219 specific antibodies were produced using an internal (RNF219-A) or C-terminal (RNF219-B) peptide by Abnova (Taiwan). Secondary antibodies were from Cell Signaling Technology (goat anti-mouse IgG HRP-linked, #7076 and goat anti-rabbit IgG HRP-linked, #7074).

### Protein sequence alignment

Amino acid sequence alignment was performed with Clustal omega tool.

### E3 ubiquitin ligase activity

20 ng FH-RNF219 or FH-RNF219-CG, purified with M2-FLAG agarose and peptide eluted, from transfected HEK293T cells under high salt conditions (0.5 M NaCl) was used in a 20 µl reaction following manufacturer instructions (BML-UW9920-0001 ENZO Life Sciences) with UBCH5a as E2 enzyme.

### Immunofluorescence

Immunofluorescence was performed essentially as previously described^35^. For 293T cells, prior seeding, coverslips (0111520, Marienfeld) were incubated in 0.01% poly-l-lysine (Sigma) for 15 min at room temperature, then washed three times with PBS. Upon harvest, coverslips were washed with PBS, fixed with 3% PFA/2% sucrose in PBS for 10–15 min at room temperature (RT), washed again in PBS and subsequently incubated in cold permeabilization buffer (20 mM Tris (pH 7.4), 50 mM NaCl, 0.5% Triton X-100, 3 mM MgCl2, 0.3 M sucrose) for 5 min at room temperature. After washes in PBST (PBS, 0.1% Tween-20), coverslips were incubated 30 min at 37 °C with primary antibody, washed three times with 1 ml PBST, followed by secondary antibody diluted in PBST. Finally, coverslips were washed again three times with 1 ml PBST and mounted on slides with DAPI containing Vectashield (H-1200, Vector Laboratories).

### Purification of RNF219-associated complexes and mass spectrometry

RNF219 complexes were purified from Dignam S100 extracts^36^ derived from 2 × 10^9^ HeLa S3 cells stably expressing RNF219 fused to a FLAG and an HA tag at the C-terminus (FHA-RNF219) by two-step affinity chromatography, according to a standard method^37^. 5% of FLAG and HA immunoaffinity purified FHA-RNF219 or mock immunoprecipitations (IP) from four liters of culture were resolved on SDS-PAGE and stained with the Silverquest kit (Invitrogen). The remainder of the eluate was stained with colloidal blue (Invitrogen) (Table S2 bottom). Individual coomassie stained bands, or for closely migrating bands regions of the gel, were excised and subsequently analyzed by tandem mass spectrometry at the Harvard Medical School Taplin Biological Mass Spectrometry facility, Boston, MA.

### Whole-cell extract preparation and immunoprecipitation

All steps were performed at 4 °C. β-Mercaptoethanol and phenylmethylsulfonyl fluoride (PMSF) were added to cold solutions prior to use. Following harvest, cells were washed with cold PBS and then resuspended in 10 cell pellet volumes of TETN-150 buffer (20 mM Tris–HCl (pH 7.4), 0.5 mM EDTA, 0.5% Triton X-100, 150 mM NaCl, 2 mM MgCl2, 5 mM β-mercaptoethanol, 0.5 mM PMSF) and incubated for 30 min with rotation. Extracts were cleared by centrifugation at 18,000*g* for 20 min and transferred to fresh tubes. Antibodies were added for 3 h before 45 min incubation with protein A or G beads (Dynabeads 10002D, 10003D from Thermofisher scientific, or agarose beads sc-2001, sc-2001 from Santa Cruz Biotechnology) for rabbit or mouse IgG respectively. After 3 washes in lysis buffer, FLAG-IPs were eluted with FLAG peptide at 0.2 mg/ml in lysis buffer at 4 °C for one hour. IPs with other antibodies were eluted by incubation in SDS loading buffer for 3 min at 95 °C.

### Luciferase assay

For each well of a six-well plate, HeLa and HEK293T cells were seeded 24 h prior to transfection. Cells were co-transfected with 100 ng of Renilla-5BoxB reporter^38,39^ and 25 ng of Firefly control reporter, along with either N-terminally tagged λN-peptide and HA tagged protein expression plasmid (NHA-LacZ (25 ng), NHA-RNF219 (600 ng), NHA-RNF219-Id (900 ng), NHA-RNF219-Cd (200 ng) NHA-CNOT7 (200 ng), NHA-CNOT1-R (200 ng), FHA-RNF219 (500 ng) plasmids, using Fugene (Promega) for HeLa or calcium phosphate (Sigma) for HEK293T. Cells were harvested 24 h after transfection. Luciferase activities were analyzed individually with the Promega E151A substrate for Firefly and the Renilla-Glo Luciferase Assay System with E2720 substrate for Renilla.

### Statistical analysis

Unpaired two-tailed Student’s *t* test were used to measure the statistical significance of the differences of the means (n = 3) between conditions.

### Quantitative RT–PCR (qRT–PCR) and poly(A) tail length analysis

Total RNA was extracted using TRIzol (Life Technologies) and digested with RQ1 DNAse (M6101 Promega) following manufacturer’s instructions. For reverse transcription, 0.5 µg of RNA was heated for 5 min (min) at 65 °C and cDNA generated using SuperScript III (Life Technologies) and random primers for 1 h (h) at 50 °C, followed by heat inactivation for 15 min at 70 °C. qPCRs (for primers, see Supplemental Table S1) were performed with SYBR Green master mix (Ozyme) using a LightCycler 480 (Roche) and incubated for 2 min at 95 °C with 35 cycles of 10 s (sec) at 95 °C, 25 s at 60 °C, and 25 s at 72 °C. Data were normalized to GAPDH and analyzed using the 2^−ΔΔCT^ method^40^. Poly(A) tail length was analyzed using the ePAT method^41^. Briefly, 1 µg of total RNA was incubated with 5 μM oligo-(dT)-anchor (5′GCGAGCTCCGCGGCCGCGTTTTTTTTTTTT3′) and 5 U of Klenow polymerase (New England Biolabs) for 1 h at 37 °C for template extension of the poly(A) tail, followed by reverse transcription using 200 U of SuperScript III (Life Technologies) for 1 h at 55 °C. Poly(A) tails were amplified by PCR using a gene-specific forward primer and the oligo-(dT)-anchor and migration of PCR product performed on 3% UltraPure Agarose 1000 gel (Life Technologies).

### Polyribosome purification

All steps were performed on ice or in a cold room. β-mercaptoethanol, RNAse inhibitor and phenylmethylsulfonyl fluoride (PMSF) were added to cold solutions prior to use. Cells were treated with 0.1 mg/ml cycloheximide (CHX) at 37 °C for 5 min prior harvesting. Following harvest, cells grown in 6 cm plates were washed twice with cold PBS containing 0.1 mg/ml CHX and frozen on dry ice or directly processed.

Per 6 cm plate, frozen cell pellets were thawed by resuspension in 200 µl lysis buffer A (20 mM Tris–HCl pH 7.4, 100 mM KCl, 10 mM MgCl_2_, 0.2 mg/ml heparin, 0.1 mg/ml CHX, 1% Triton, 2 mM β-mercaptoethanol, 0.5 mM PMSF, 20 U/ml SUPERaseIn (Ambion) and incubated 15 min on ice. After 15 min centrifugation at 16,100*g*, 170 µl supernatant was loaded on a 4.5 ml 15–50% sucrose gradient prepared in buffer B (20 mM Tris–HCl pH 7.4, 50 mM KCl, 10 mM MgCl2, 0.1 mg/ml CHX, 2 mM β-mercaptoethanol, 0.5 mM PMSF, 4 U/ml SUPERaseIn (Ambion). Briefly, 0.5 ml 50% and 1 ml of each 40%, 30%, 20% and 15% sucrose solution was layered sequentially and diffused at 4 °C for 16 h to obtain the gradient.

Gradients were spun in a SW55Ti rotor at 46,000 rpm for 70 min. Fractions were manually collected from the top of the gradient. 1% SDS was added to fractions of interest prior to RNA extraction with acidic phenol chloroform (Ambion 9720) following manufacturer instructions. RNA samples were treated with RQ1 DNAse (Promega), heat inactivated, followed by reverse transcription using SuperScript IV (Life technologies) and qPCR analysis, according to manufacturer instructions.

### RNA-seq procedure and sequences analysis

HeLa cells were transfected with either a control siRNA (siSCR) or a siRNA against RNF219 (CDS) in two replicates each. RNA was extracted with TRIzol (Life Technologies) reagent according to the manufacturer’s instructions. RNA samples were then treated with DNase I, extracted again with TRIzol (Life Technologies) and precipitated.

Ribosomal RNA depletion and RNA-Seq library preparation were performed at Fasteris SA (Plan-les-Ouates, Switzerland) using TruSeq stranded mRNA kit (Illumina). RNAseq samples were sequenced using Illumina HiSeq High-Output (HO), single-end, 50 bp reads at Fasteris SA.

Raw RNA-sequencing reads were trimmed of adapters and low-quality regions using Trim Galore! version 0.6.4. Reads were then aligned to the human reference genome, Ensembl assembly GRCh38.p13 release 101 using HISAT2 version 2.2.1. HTSeq version 0.12.4 was used for gene-level quantification of reads using the corresponding Ensembl annotation file (Homo_sapiens.GRCh38.101.gtf). Normalization and differential expression analysis were performed using DESeq2. Gene set enrichment analysis was done using the R package fgsea with the GSEA curated GO biological process gene list version 7.2. The code used for analyzing RNA-sequencing reads is available at: https://github.com/lirpa0/RNF219_DE/blob/main/RNAseq_analysis_code_9_9_21.md.

## Results

### Characterization of RNF219 a RING dependent ubiquitin ligase

**Figure 1:**
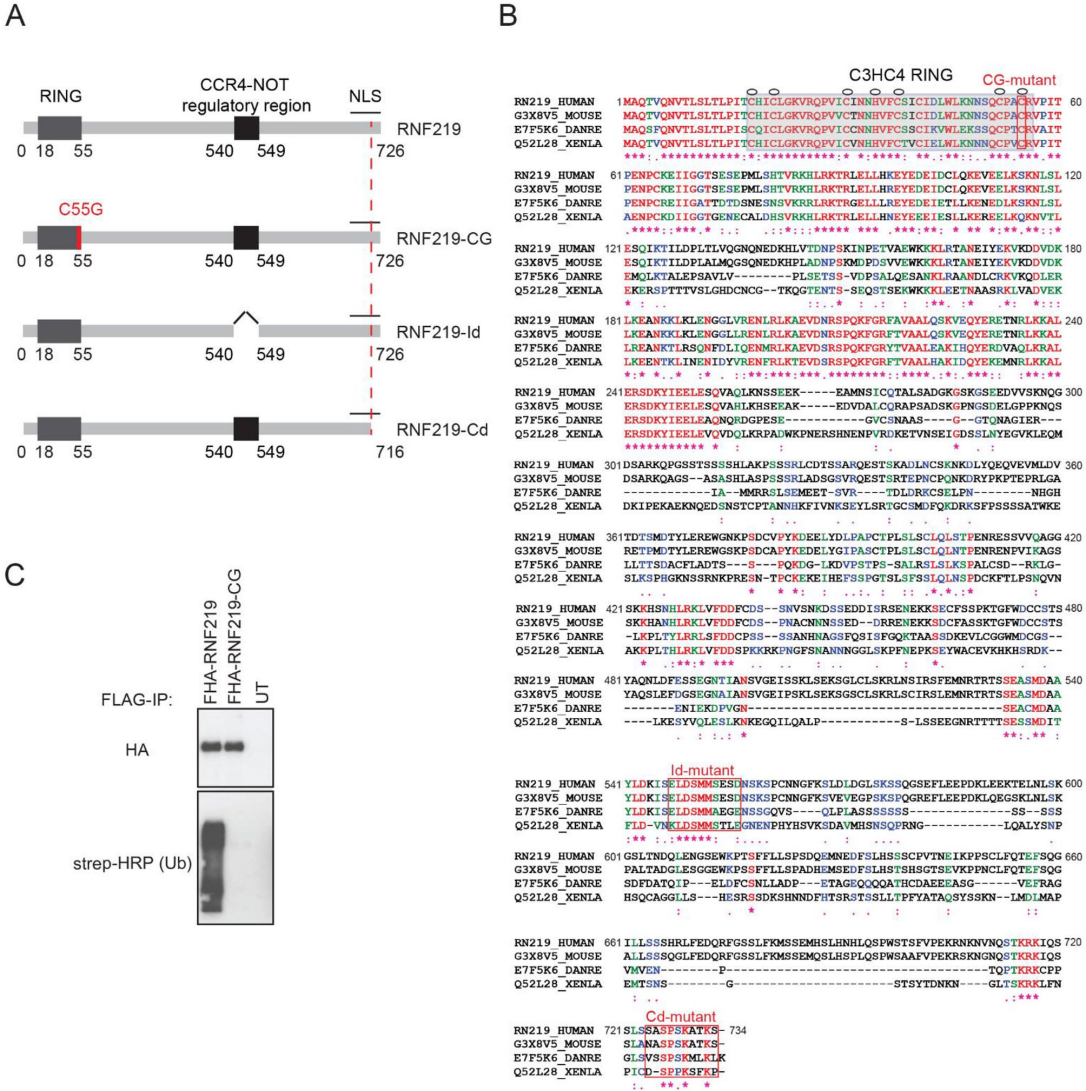
RNF219 is a RING dependent ubiquitin ligase. (**A**) Schematic representation of the RNF219 protein and mutants used in this study. (**B**) Sequence alignment of the human hRNF219 protein and its *Mus musculus*, *Xenopus laevis* and *Danio rerio*, orthologs. Amino acid sequences were aligned with the Prabi CLUSTALW tool. Identical residues are annotated with an asterisk (*) and coloured in red, whereas similar residues are annotated with two (:) and coloured in green and less similar residues are annotated with one dot (.) and coloured in blue. One conserved RING domain of C3HC4 type was predicted in the N-terminus of all the conserved alignments (highlighted in grey); Zinc coordinating residues are marked with a circle. Mutated residues and corresponding mutants are highlighted by a red box. (**C**) RNF219 but not the RING domain mutant can form poly-ubiquitin chains in an in vitro assay. FHA-RNF219 and FHA-RNF219-CG (cysteine 55 was replaced by a glycine) affinity purified under high salt condition with FLAG antibody and peptide eluted from transfected 293T cells, were incubated with E1 enzyme, UBCH5a, and biotinylated ubiquitin (lanes 1 and 2, respectively). The third lane contains the purification from untransfected cells as negative control. Ubiquitin was detected with HRP conjugated streptavidin (Strep-HRP).

We initially identified RNF219 as a substoichiometric interactor of the HIV-1 Tat transcription factor (Personal communication)^42^. RNF219 is a 726 amino acid protein containing at its N-terminus a 37-amino acid length C3HC4 RING finger domain (Fig. 1A,B)^43^. According to the Ensembl Compara database (PMID: 26896847), RNF219 is present in a single copy in the genome of most placental mammals. Orthologues are found in the genomes of many other vertebrates (bird, reptiles and fish) but not among other metazoans (Fig. 1B). Immuno-localization of FLAG-HA tagged RNF219 (FHA-RNF219) expressed in U2OS cells shows that this protein is found in both the cytoplasm (60% of cells n = 110, Fig. S1A) and the nucleus (40% of cells n = 110, Fig. S1A). Of note, RNF219 was not found in both compartments simultaneously.

To better characterize RNF219, we generated two polyclonal antibodies (αRNF219-A and αRNF219-B) directed against human RNF219, and two RNF219 CRISPR KO cell lines (HEK293T SG1-C and HeLa SG1-C) (Fig. S1B-C). Immunoblots performed on HeLa cell extracts showed that αRNF219-A specifically recognizes a 85 kDa protein that disappeared upon RNF219 depletion using two different small interfering RNAs (siRNAs), targeting RNF219 coding sequence (CDS) or 3′UTR (UTR), respectively (Fig. S1B). The second antibody (αRNF219-B) was used to immuno-precipitate endogenous RNF219 (Fig. S1D, 2C, S2A,B). A third antibody, αRNF219-C (from Bethyl laboratory) was used to immuno-precipitate endogenous RNF219 in order to confirm the data obtained with αRNF219-B (Fig. S2B).

Since RNF219 contains a RING finger domain (Fig. 1B), found in many E3 ubiquitin ligases, we tested its ability to catalyze the formation of polyubiquitin chains. An in vitro E3 ubiquitin ligase assay was performed with FHA-RNF219, immunoprecipitated from 293T cell extract under high salt conditions, in the presence of E1, E2 (UBCH5a) enzymes and biotinylated ubiquitin. Wild-type FHA-RNF219, but not a mutant form containing a point mutation in the RING domain (cysteine 55 changed to a glycine; RNF219-CG), was able to form polyubiquitin chains in this biochemical assay (Fig. 1C). This result suggests that RNF219 is a RING domain dependent ubiquitin ligase, consistent with a previous report^44^.

### RNF219 associates with the CCR4-NOT complex

**Figure 2:**
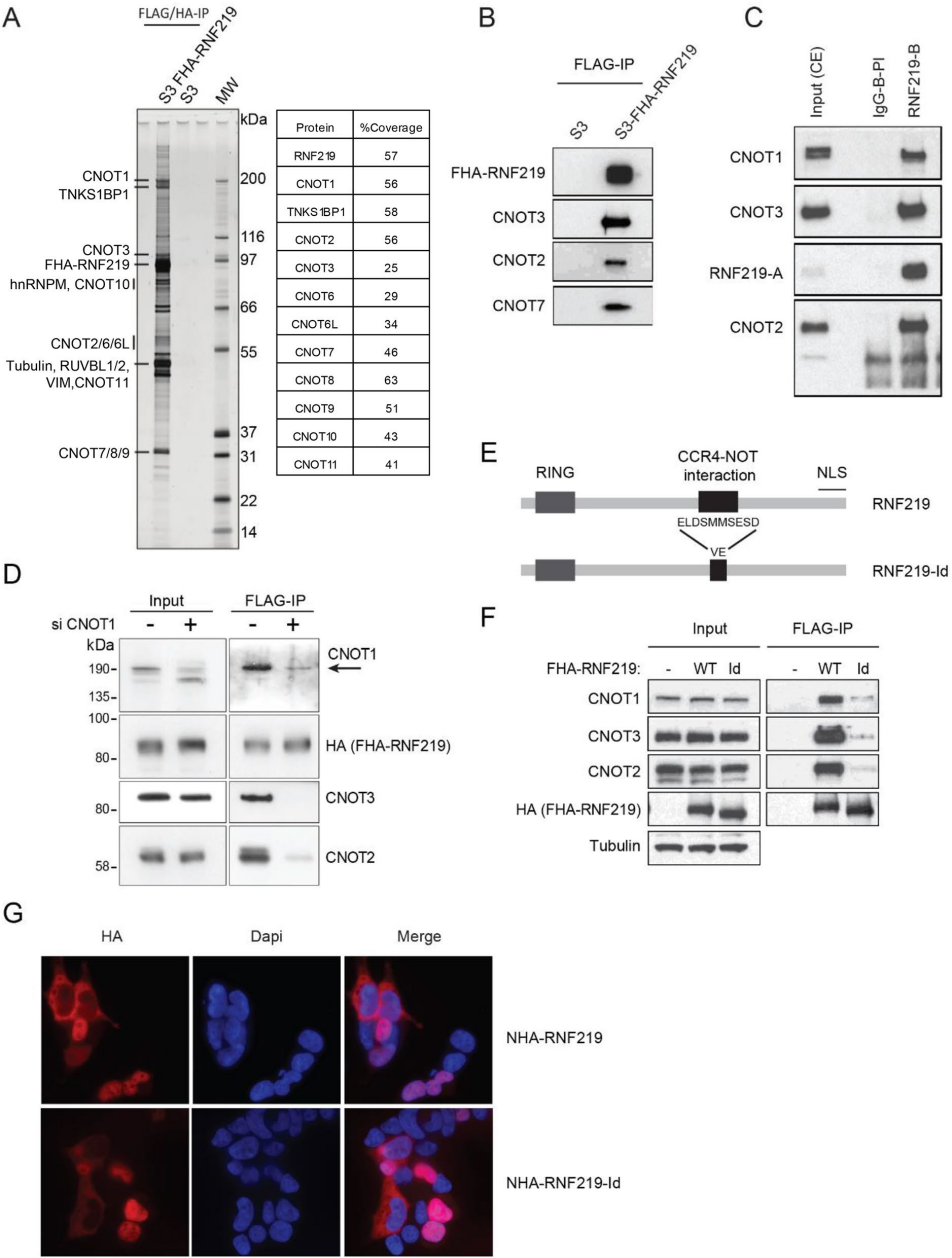
RNF219 binds to the CCR4-NOT complex. (**A**) (Left) FLAG and HA tandem affinity chromatography was performed on cytoplasmic S100 extracts prepared from HeLa S3 cells stably expressing FHA-RNF219 (S3-FHA-RNF219) or non-transduced HeLa S3 cells. Proteins were resolved by SDS-PAGE and visualized by silver staining. (Right) The identity of FHA-RNF219-associated proteins was determined by tandem mass spectrometry and percent peptide coverage by amino acid count for CCR4-NOT subunits detected are listed. (**B**) FLAG-immuno-precipitations from S3-FHA-RNF219 cell extracts confirm that FHA-RNF219 is associated with a number of CCR4-NOT subunits as determined by mass spectrometry. The presence of CCR4-NOT subunits CNOT3, CNOT2, CNOT7 in the IPs was analyzed by immunoblotting (IB). (**C**) Endogenous RNF219 was immuno-purified from HeLa cell extracts using a specific antibody against RNF219 (RNF219-B) or pre-immune IgG control (IgG-B-PI). The presence of the CCR4-NOT subunit CNOT1, CNOT2 and CNOT3 in the IP was analyzed by IB. RNF219 was detected with RNF219-A antibody (long exposure is shown in Fig. S2B). (**D**) RNF219 interaction with NOT module components CNOT2 and CNOT3 is CNOT1 dependent. FHA-RNF219 expressing HeLa cells were transfected with siRNA against CNOT1 (+) or control siRNA (−). IPs were performed using anti-FLAG agarose beads and were blotted for the indicated proteins. The arrow indicates the expected CNOT1 band. (**E**) Schematic representation of RNF219-Id, a CCR4-NOT complex interaction defective RNF219 allele. Amino acids 540–549 (ELDSMMSESD) were replaced by VE. (**F**) RNF219-Id interacts with the CCR4-NOT complex very inefficiently. 293T cells were transfected with the indicated constructs. Whole cell extracts were immuno-purified with anti-FLAG agarose beads and eluates immunoblotted with the indicated proteins. (**G**) RNF219-Id localizes both in the nucleus and in the cytoplasm similarly to WT RNF219. Immuno-fluorescence was performed with anti-HA antibody on HEK293T cells transfected with the indicated constructs.

We purified RNF219 protein complexes to address its cellular function. TAP-tag experiments^37^ were performed using cytoplasmic S100 extract^36^ from HeLa S3 cells stably expressing FLAG and HA-tagged RNF219 (FHA-RNF219). Immunoprecipitated material was analyzed by tandem mass spectrometry. Remarkably, all subunits of the CCR4-NOT except CNOT4 were identified with the high peptide coverage (Fig. 2A and highlighted in yellow in Table S2). Of note, we observed robust peptide coverage measured for the different CCR4-NOT deadenylase subunits CNOT6, CNOT6L, CNOT7 and CNOT8, suggesting that RNF219 can stably associate with CCR4-NOT complexes containing either subunit. Our finding is consistent with a recent studies, which independently describe the interaction between RNF219 and CCR4-NOT^32,33^.

CNOT3, CNOT2 and CNOT7 co-purification with FHA-RNF219 were confirmed by FLAG immunoprecipitation followed by immunoblotting with antibodies directed against these CCR4-NOT subunits (Fig. 2B). Importantly, endogenous CCR4-NOT subunits co-immunoprecipitated with endogenous RNF219 pulled down using two different antibodies (RNF219-B and RNF219-C) for IP (Fig. 2C, Fig. S2A,B). To control that the interaction is not due to a protein non-specifically pulled down with RNF219-B antibody, immunoprecipitation was performed with RNF219-B antibody on RNF219-Crispr knockout cells transfected or not with a plasmid containing RNF219 cDNA (Fig. S1D; respectively RNF219 and Mock). This experiment shows that CNOT2 co-immunoprecipitates with RNF219-B antibody only in RNF219 knockout cells reconstituted with RNF219 (Fig. S1D) which confirms the specificity of RNF219 interaction with at least one subunit of the complex. Additionally, immunoprecipitation of endogenous CNOT3 and CNOT1 also co-immunoprecipitate RNF219, further confirming our results (Fig. S2A).

As CNOT1 is the core subunit of the CCR4-NOT complex^11–13,45^, we next hypothesized that the depletion of CNOT1 could abrogate RNF219 association with subunits of the complex. As predicted, upon siRNA-mediated depletion of the CNOT1 scaffold, RNF219 co-precipitation with the other components of the CCR4-NOT complex, CNOT2 and CNOT3, was strongly reduced (Fig. 2D).

To clone a CCR4-NOT interaction defective allele, we truncated RNF219 protein in multiple fragments until interaction with CCR4-NOT was lost (Fig. S2C,D). RNF219 was first truncated in six large fragments with a truncation step of 121 amino acids (represented by F1 to F6 in Fig S2C). The F5 fragment failed to interact with at least three CCR4-NOT subunits (CNOT1, CNOT2, CNOT3; Fig. S2C). We further truncated F5 into four fragments with a truncation step of 10 amino acids (represented by F5F, F5G, F5H, F5I in Fig. S2D). CCR4-NOT interaction was lost with the F5G fragment (Fig. S2D). This led to the identification of a minimal domain necessary for RNF219 binding to the CCR4-NOT complex (Fig. 2E,F; Fig. S2D). Replacement of the amino acids ELDSMMSESD by VE in the middle part of RNF219 strongly reduces RNF219 interaction with CNOT1, CNOT2 and CNOT3 (Fig. 2F). We named this interaction defective mutant RNF219-Id (Figs. 1A, 2E,F). Of note, the interaction between RNF219 and the CCR4-NOT complex is RNAse resistant under conditions dissociating the 7SKsnRNP as previously reported^46,47^ (Fig. S2E), indicating that the CCR4-NOT and RNF219 interaction is not mediated by RNA. However, RNA may play a role during the formation of the complex. Finally, we showed by immuno-localization that RNF219-Id, similarly to WT RNF219, is present either in the nucleus or in the cytoplasm (Fig. 2G).

Altogether, our data reveal physical interaction of RNF219 with the CCR4-NOT complex through purification of exogenously expressed, tagged proteins, and importantly also with purification of endogenous proteins using several antibodies. RNF219 binding requires the CNOT1 scaffold but not RNA for its association to the NOT module. We do not rule out the possibility that RNF219 may directly bind a CCR4-NOT subunit not tested or CCR4-NOT recruiting proteins present in the ms/ms data with high score.

### RNF219 represses the translation of a reporter mRNA

**Figure 3:**
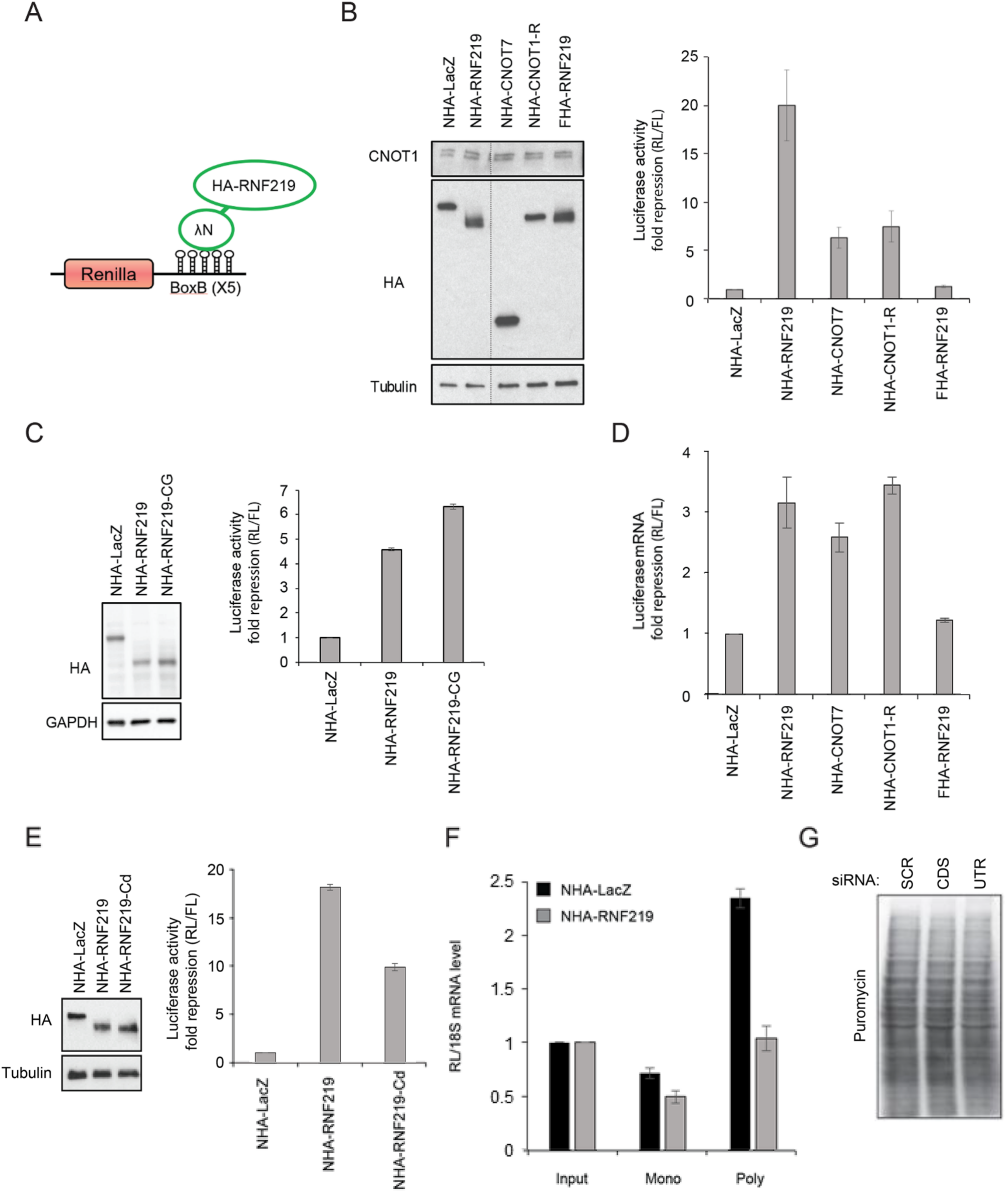
RNF219 affects the translation of a targeted mRNA. (**A**) Schematic representation of the Renilla Luciferase (RL) reporter mRNA, containing the Renilla luciferase gene and five 19-nt BoxB hairpins sequences at its 3′UTR. Recruitment of HA-tagged protein to the 3′UTR is mediated by the fused λN-peptide, which has a high affinity for the BoxB sequence. (**B**) Recruitment of RNF219 to the 3′UTR mRNA of the RL reporter inhibits its expression. RL activity was determined in the indicated conditions (NHA-LacZ, NHA-RNF219, NHA-CNOT7, NHA-CNOT1-R, FHA-RNF219). (Right) Renilla Luciferase (RL) activity was normalized on Firefly luciferase (FL) activity expressed from a plasmid not containing BoxB sequences, co-transfected with that of Renilla. RL repression levels are relative to that of the control NHA-LacZ set to 1. Error bars represent SD, n = 3. (Left) Protein levels of NHA-fusion proteins in transfected 293T cells was analyzed by immunoblotting. (**C**) Ubiquitin ligase activity of RNF219 is not necessary for its repressive role. RL activity was determined in the indicated conditions (NHA-LacZ, NHA-RNF219, NHA-RNF219-CG). (Right) Renilla Luciferase (RL) activity was normalized as in (**B**). Error bars represent SD, n = 3. (Left) Protein levels of NHA-fusion proteins, in HeLa cells transfected with the corresponding plasmids, were analyzed by immunoblotting. (**D**) Recruitment of RNF219 to the 3′UTR mRNA of the RL reporter leads to decreased reporter mRNA level. Total RNA from the indicated conditions (NHA-LacZ, NHA-RNF219, NHA-CNOT7, NHA-CNOT1-R, FHA-RNF219) was extracted as described in the “Methods”. RT-QPCR was performed using primers specific from both RL and FL reporters to quantify their respective mRNA level (the RT-QPCR primers used are indicated in Table S1). RL mRNA level was normalized on FL mRNA level. RL repression levels are relative to that of the control NHA-LacZ set to 1. Error bars represent SD, n = 3. (**E**) RNF219 mutant (RNF219-Cd) that resides predominantly in the cytoplasm also represses the RL expression. (Right) RL activity was determined in the indicated conditions (NHA-LacZ, NHA-RNF219, NHA-RNF219-Cd). Renilla Luciferase (RL) activity was normalized as in (**B**). Error bars represent SD, n = 3. (Left) Protein levels of NHA-fusion proteins in 293T cells transfected with the corresponding plasmids were analyzed by immunoblotting. (**F**) RL mRNA level relative to FL mRNA level in the monoribosomal (Mono, fraction 4 in Fig. S3B) and polyribosomal fractions (Poly, fraction 9 in Fig. S3B) of extracts from 293T cells transfected with NHA-LacZ or NHA-RNF219. mRNA levels normalized to 18S mRNA levels were quantified by RT-QPCR and shown relative to Input set to 1. Error bars represent SD, n = 2. (**G**) RNF219 does not affect global translation. 24 h after siRNA treatment (two different siRNA against RNF219: CDS and UTR; Fig. S1B) a 30-min puromycin pulse is analysed by immunoblot using an anti-puromycin antibody. The two siRNA against RNF219 show no difference in global puromycin incorporation compared to the control (SCR).

To characterize RNF219 functionally, we asked whether RNF219 plays a role in post-transcriptional regulation of mRNA, a well-established CCR4-NOT function. We set up a classical λNpeptide/BoxB RNA tethering reporter assay to address this question (described and characterized in^38,39,48,49^). Briefly, in this assay, a λNpeptide-tagged candidate protein (here RNF219) is targeted to a Renilla luciferase (RL) reporter mRNA through binding of the λNpeptide to BoxB sites present in the reporter 3′UTR (Fig. 3A).

HEK293T cells were co-transfected with the RL reporter and either λN and HA-tagged-LacZ (control NHA-LacZ), or -RNF219 construct (NHA-RNF219; Fig. 3B,C,E). Subsequently, the RL reporter expression was assessed by measuring the Renilla luciferase activity relative to a Firefly luciferase reporter not bound by the transfected protein (RL/FL ratio). Interestingly, tethering RNF219 strongly repressed the RL reporter mRNA expression compared to the NHA-LacZ control (RL/FL ratio: RNF219 *M* = 0.307 *SD* = 0.0624, LacZ *M* = 5.97 *SD* = 0.276; two tailed *t* test: *t*(2.2) = 34.5, *p* < 0.001; Fig. 3B). RNF219-mediated repression was higher than repression mediated by the CCR4-NOT subunit CNOT7 and the CNOT1-R fragment^26^ (Fig. 3B) previously described^26,28^. Both NHA-CNOT7 and NHA-CNOT1-R displayed significantly lower mean RL/FL ratios than the NHA-LacZ control (CNOT7 *M* = 0.961 *SD* = 0.184; two tailed *t* test compared to NHA-LacZ: *t*(3.49) = 26.1, *p* < 0.0001; and CNOT1R *M* = 0.825 *SD* = 0.189; two tailed *t* test compared to NHA-LacZ: *t*(3.53) = 26.6, *p* < 0.0001), as expected. Strikingly, NHA-RNF219 exhibited an even lower mean RL/FL ratio than NHA-CNOT7 and NHA-CNOT1-R (RNF219 compared to CNOT7: two tailed *t* test: t(2.45) = −  5.82, p < 0.05; compared to CNOT1R: two tailed *t* test: t(2.43) = − 4.51, p < 0.05). Of note, when FHA-RNF219 was not fused to λN-peptide it did not substantially repress the reporter, with a fold change repression of only 1.32 relative to the LacZ control (FHA-RNF219 *M* = 4.57 *SD* = 0.555; two tailed *t* test: *t*(2.94) = 3.92, *p* < 0.05; Fig. 3B, last bar). This suggests that NHA-RNF219 acts in cis in the tethering assay.

Next, we investigated whether the ubiquitin-ligase activity we characterized (Fig. 1C) was necessary for RNF219 to repress the RL reporter. Hence, we tested the RING-mutated version of RNF219 described in Fig. 1C (RFN219-CG) in the tethering assay. Surprisingly, RNF219-CG was able to repress the reporter even more strongly than WT RNF219 (RL/FL ratio NHA-RNF219-CG *M* = 0.169 *SD* = 0.00875, NHA-RNF219 *M* = 0.233 *SD* = 0.00621; two tailed *t* test: *t*(3.61) = − 10.4, *p* < 0.001; Fig. 3C). This observation indicates that the ubiquitin ligase activity of RNF219 is not necessary for repression. The ubiquitin ligase activity may however be required for steps bypassed in the tethering assay, such as binding to specific target RNAs. Indeed, RNF219 has a much stronger repressive effect when tethered to the reporter RNA (Fig. 3B), suggesting that in vivo this protein may be directed to specific targets.

### RNF219-mediated repression is not solely due to mRNA degradation

CCR4-NOT tethering to reporter mRNA classically leads to RNA degradation^13^. Thus, we monitored the RL reporter mRNA level in these cells to test whether repression of luciferase activity was due to a decrease in mRNA quantity (Fig. 3D). Expectedly, tethering CNOT7 or CNOT1-R induced a 2.6 to 3.5-fold decrease in mRNA level (RL/FL mRNA levels: CNOT7 *M* = 9.34 *SD* = 0.101, CNOT1-R *M* = 7.0 *SD* = 0.342, LacZ *M* = 24.0 *SD* = 2.00; two tailed *t* test: *t*(2.01) = − 12.7, *p* < 0.01 comparing LacZ and CNOT7; *t*(2.12) = − 14.5 *p* < 0.01 comparing LacZ and CNOT1-R). Tethering NHA-RNF219 decreased mRNA levels by a similar magnitude (3.14 fold; RL/FL mRNA levels NHA-RNF219 *M* = 7.68 *SD* = 0.40; two tailed *t* test comparing RNF219 and CNOT7: *t*(2.25) = − 6.91, *p* < 0.05); comparing RNF219 and CNOT1-R: *t*(3.90) = 2.23, *p* > 0.05). That RNF219 has similar repressive effects as CNOT7 and CNOT1-R at the mRNA level (Fig. 3D) stands in contrasts with the much stronger repressive effects of RNF219 when compared to CNOT7 and CNOT1-R at the level of luciferase activity (Fig. 3B). This suggests that mRNA degradation alone does not explain the full RNF219-mediated RL reporter activity repression.

We observed that RNF219 localizes either in the nucleus or in the cytoplasm (Fig. S1A). Thus to investigate the significance of RNF219 localization for its repressive activity, we tested a RNF219 mutant (RNF219-Cd) that resides predominantly in the cytoplasm (Fig. 1A; Fig. S3A). We found that RNF219-Cd also represses the RL expression compared to the LacZ control (RL/FL ratio: NHA-RNF219-Cd *M* = 0.388 *SD* = 0.0092, NHA-LacZ *M* = 3.83 *SD* = 0.0516; two tailed *t* test: *t*(2.13) = − 114, *p* < 0.0001), indicating that this activity is in part a cytoplasmic process (Fig. 3E) and cannot be explained by sequestration of mRNA in the nucleus as the only mechanism. However, NHA-RNF219-Cd is not fully active compared to NHA-RNF219 (*M* = 0.212 *SD* = 0.00613; two tailed *t* test comparing NHA-RNF219 and NHA-RNF219-Cd: *t*(3.48) = − 27.6, *p* < 0.0001), indicating that nuclear RNF219 function may also participate in its repressive activity.

### RNF219 inhibits mRNA translation

To further explore the molecular mechanism by which RNF219 represses the RL expression, we asked whether RNF219 affects mRNA translation. To answer this question, we purified poly-ribosomes from cells transfected with the RL reporter and NHA-LacZ or NHA-RNF219 constructs. Mono and poly-ribosomal fractions were identified by levels of ribosomal protein subunit RPL7a, optical density at 254 nm and distribution of 18S and 28S RNA (Fig. S3B). RL reporter mRNA was significantly less abundant in the poly-ribosomal fraction of cells expressing NHA-RNF219 compared to cells expressing NHA-LacZ (RL/FL mRNA levels normalized to 18S: NHA-RNF219 *M* = 1.04 *SD* = 0.113, NHA-LacZ *M* = 2.35 *SD* = 0.0919; two tailed *t* test: *t*(1.92) = 12.7, *p* < 0.01; Fig. 3F). We verified that this effect was not attributable to a global defect in translation by comparing puromycin incorporation in control (siSCR) or RNF219 (siCDS and siUTR) depleted cells^50^; Fig. 3G; Fig. S3C, S1B). No major difference was detected in global translation activity between both conditions, suggesting that RNF219 is involved in translational repression of specific mRNAs.

### RNF219-mediated mRNA translation repression is CCR4-NOT-dependent

**Figure 4:**
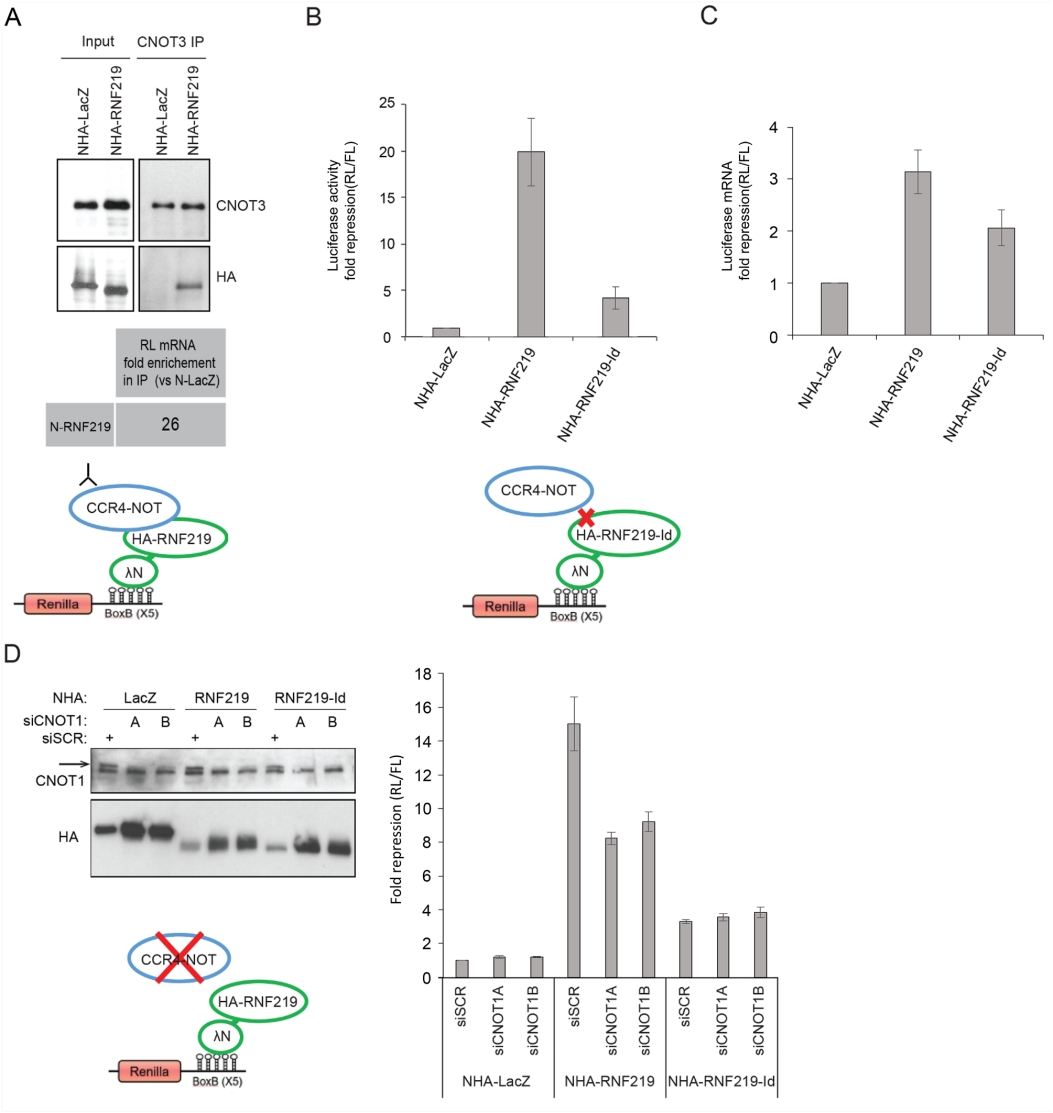
RNF219 mediated translational repression is CCR4-NOT dependent. (**A**) CNOT3 interacts with RNF219 tethered to the mRNA reporter. RNA-IPs were performed on extracts of cells transfected with the RL reporter and NHA-LacZ or NHA-RNF219 using CNOT3 antibody. The level of Renilla mRNA in the CNOT3-IP was highly enriched in NHA-RNF219 transfected cells as compared to NHA-LacZ transfected cells, as quantified by RTPCR. Results representative of two replicates are shown. (**B**) RNF219 interaction with the CCR4-NOT complex is necessary for RFN219 to repress RL reporter translation efficiently. (Top) RL activity was determined in the indicated conditions (NHA-LacZ, NHA-RNF219 and NHA-RNF219-Id). RL activity repression was determined as in Fig. 3B. Error bars represent SD, n = 3. (Bottom) Protein levels of NHA-LacZ, NHA-RNF219, NHA-RNF219-Id in HeLa cells transfected with the corresponding plasmids were analyzed by immunoblotting. (**C**) mRNA levels of samples in (**B**) were quantified as in Fig. 3D. Error bars represent SD, n = 3. (**D**) Knock down of CCR4-NOT scaffold CNOT1 affects NHA-RNF219 mediated repression. RL activity was determined in the indicated conditions (NHA-LacZ, NHA-RNF219 and NHA-RNF219-Id) in control cells (siSCR) or in siCNOT1 depleted cells (siCNOT1A and siCNOT1B are two siRNA targeting different regions of CNOT1 mRNA). (Right) RL activity repression was determined as in Fig. 3B. RL repression levels are relative to that of the control NHA-LacZ in the siSCR cells, set to 1. Error bars represent SD, n = 3. (Left) Protein levels. The arrow indicates the expected CNOT1 band. The lower is a non-specific band as it does not disappear in siCNOT1 treated cells.

To determine if RNF219 translation inhibition capacity was CCR4-NOT-dependent, we first verified that NHA-RNF219 recruited CCR4-NOT to the reporter mRNA in the tethering assay (Fig. 4A). Endogenous CNOT3 immunoprecipitation showed that CNOT3 specifically co-purified with the reporter mRNA, in presence of NHA-RNF219 compared to NHA-LacZ, consistent with the recruitment of CCR4-NOT to the mRNA by NHA-RNF219 (Fig. 4A).

Second, we tested the CCR4-NOT interaction defective allele (NHA-RNF219-Id) in the tethering assay. This mutant was significantly less efficient in repressing the RL reporter activity compared to NHA-RNF219 (RL/FL NHA-RNF219-Id *M* = 1.50 *SD* = 0.415, NHA-RNF219 *M* = 0.307 *SD* = 0.0624; two tailed *t* test: *t*(2.09) = − 4.91, *p* < 0.05) (Fig. 4B; 20-fold for RNF219 compared to less than fivefold for RNF219-Id). Of note, even though mRNA levels were slightly higher in NHA-RNF219-Id than in NHA-RNF219 (RL/FL mRNA ratio: NHA-RNF219-Id *M* = 11.8 *SD* = 1.14, NHA-RNF219 *M* = 7.68 *SD* = 0.403; two tailed *t* test: *t*(2.49) = 5.88, *p* < 0.05; Fig. 4C), this could not explain the lower repression observed with NHA-RNF219-Id (Fig. 4B). This implies that RNF219 acts at least partially through its interaction with CCR4-NOT to repress the RL reporter mRNA translation. We further confirmed this interpretation, using siRNA against CNOT1 (siCNOT1A and siCNOT1B) that abolished RNF219 association at least with part of the CCR4-NOT complex (Fig. 2D). Consistently, the RNF219 mediated translational repression of the reporter mRNA was reduced by twofold in CNOT1-depleted cells (Fig. 4D). The mean reporter activity was significantly higher for both siCNOT1a compared to the scrambled control (RL/FL ratio: NHA-RNF219 siCNOT1a *M* = 0.379 *SD* = 0.0251, NHA-RNF219 siSCR *M* = 0.208 *SD* = 0.0168; two tailed *t* test: *t*(3.49) = − 9.78, *p* < 0.001), and for siCNOT1b compared to the scrambled control (NHA-RNF219 siCNOT1b *M* = 0.34 *SD* = 0.0515; two tailed *t* test: *t*(2.42) = − 4.21, *p* < 0.05). Interestingly, CNOT1 depletion did not further reduce the residual repressive activity of RNF219-Id: there was no significant difference in mean RL/FL ratio between NHA-RNF219-Id siCNOT1a compared to the scrambled control (NHA-RNF219-Id siCNOT1a *M* = 0.879 *SD* = 0.133, NHA-RNF219-Id siSCR *M* = 0.949 *SD* = 0.103; two tailed *t* test: *t*(3.76) = 0.714, *p* > 0.05) or NHA-RNF219-Id siCNOT1b compared to the scrambled control (NHA-RNF219-Id siCNOT1b *M* = 0.82 *SD* = 0.143, two tailed *t* test: *t*(3.65) = 1.27, *p* > 0.05), suggesting that RNF219 may also function in CCR4-NOT independent repression.

These results indicate that RNF219 in complex with CCR4-NOT represses mRNA translation.

### RNF219 affects the poly(A) tail length of a targeted mRNA

**Figure 5:**
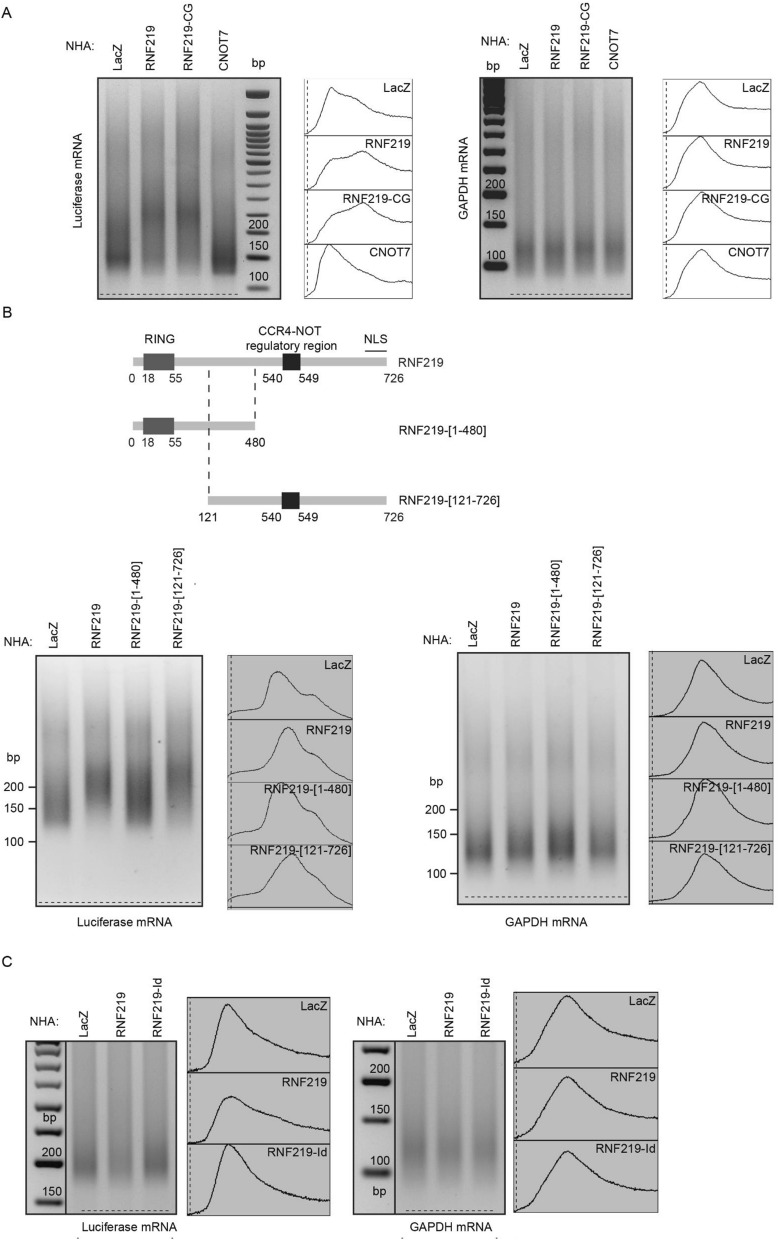
RNF219 affects the polyA tail length of a targeted mRNA. (**A**) RNF219 stabilizes the reporter mRNA poly(A) tail. ePAT assays were performed using HeLa cells expressing the RL reporter and either of the following constructs: NHA-LacZ, NHA-RNF219, NHA-RNF219-CG or NHA-CNOT7. On the left panel, a PAT primer (Luc_3; Table S1) specific to the poly(A) tail of the RL reporter gene is used. On the right panel a PAT primer specific to the poly(A) tail of the GAPDH control gene is used (Table S1). Corresponding image analyzer profiles are shown at the right of each ePAT gel. (**B**) RNF219 mRNA poly(A) tail stabilization does not depend on its RING Finger domain but depends on its interaction with the CCR4-NOT complex. ePAT assays were performed using HeLa cells expressing the RL reporter and either of the following constructs: NHA-LacZ, NHA-RNF219, NHA-RNF219-[1–480], NHA-RNF219-[121–726]. On the left panel, a PAT primer (Luc_2; Table S1) specific of the poly(A) tail of the RL reporter gene is used. On the right panel a PAT primer specific of the poly(A) tail of the GAPDH control gene is used. Corresponding image analyzer profiles are shown at the right of each ePAT gel. (**C**) RNF219 mRNA poly(A) tail stabilization depends on its interaction with the CCR4-NOT complex. ePAT assays were performed using HeLa cells expressing the RL reporter and either of the following constructs: NHA-LacZ, NHA-RNF219, NHA-RNF219-Id. On the left panel, a PAT primer (Luc_3; Table S1) specific to the poly(A) tail of the RL reporter gene is used. On the right panel a PAT primer specific to the poly(A) tail of the GAPDH control gene is used. Corresponding image analyzer profiles are shown at the right of each ePAT gel.

The CCR4-NOT complex can repress translation either through its associated deadenylation activity or through the DDX6 pathway acting at the 5′ end of mRNA^26,27^. Several CCR4-NOT associated E3 ligases have been reported to increase deadenylation activity leading to accelerated mRNA decay^51,52^. We therefore analyzed the poly(A) tail length of RNF219-tethered reporter mRNA using the extension Poly(A) Test (ePAT) assay^41,53^. As expected, tethering the CCR4-NOT subunit CNOT7 resulted in shortening of the RL reporter mRNA poly(A) tail which can be observed by the accumulation of signal at the bottom of the lanes (Fig. 5A; lane 4). RNF219 tethering had the opposite effect and led to an increased poly(A) tail length of the reporter mRNA (Fig. 5A, compare lane 1 and 2). As a control, the poly(A) tail of endogenous GAPDH mRNA, which is not targeted by lambda peptide, was not affected.

To investigate whether the impact of RNF219 on poly(A) tail length was dependent on RNF219 interaction with CCR4-NOT, we used an RNF219 interaction mutant NHA-RNF219-[1–480] (upper panel Fig. 5B and Fragment F5 in Fig. S2C) which lacks the C-terminus containing the interaction domain. This interaction mutant inefficiently represses the RL reporter expression (Fig. S4A,B). We tested the effect of the interaction mutant on the reporter mRNA poly(A) tail length (Fig. 5B). While RNF219 lengthened the reporter mRNA poly(A) tail, the interaction mutant NHA-RNF219-[1–480] did not (Fig. 5B). We also tested the effects of the NHA-RNF219-Id interaction defective allele, which is unable to recruit the CCR4-NOT complex efficiently (Fig. 2E,F). This mutant did not cause lengthening of the poly(A) tail of the reporter (Fig. 5C, compare lane 3 with lane 2). These results suggest that RNF219 interaction with CCR4-NOT is necessary for RNF219-mediated inhibition of mRNA deadenylation.

Of note, NHA-RNF219-CG, the RING domain mutant (Fig. 1C) as well as a RNF219 RING-depleted allele, NHA-RNF219-[121–726], displayed similar activity as WT RNF219, indicating that the ubiquitin ligase activity is dispensable for the effect of RNF219 on mRNA poly(A) tail lengthening in our tethering assay (Fig. 5A; lane 3, Fig. 5B; lane 4). As we previously commented (Fig. 3C), it is likely that RNF219 ubiquitin ligase activity serves at steps absent in the assay.

### Endogenous RNF219 has a role in cell cycle regulation

**Figure 6:**
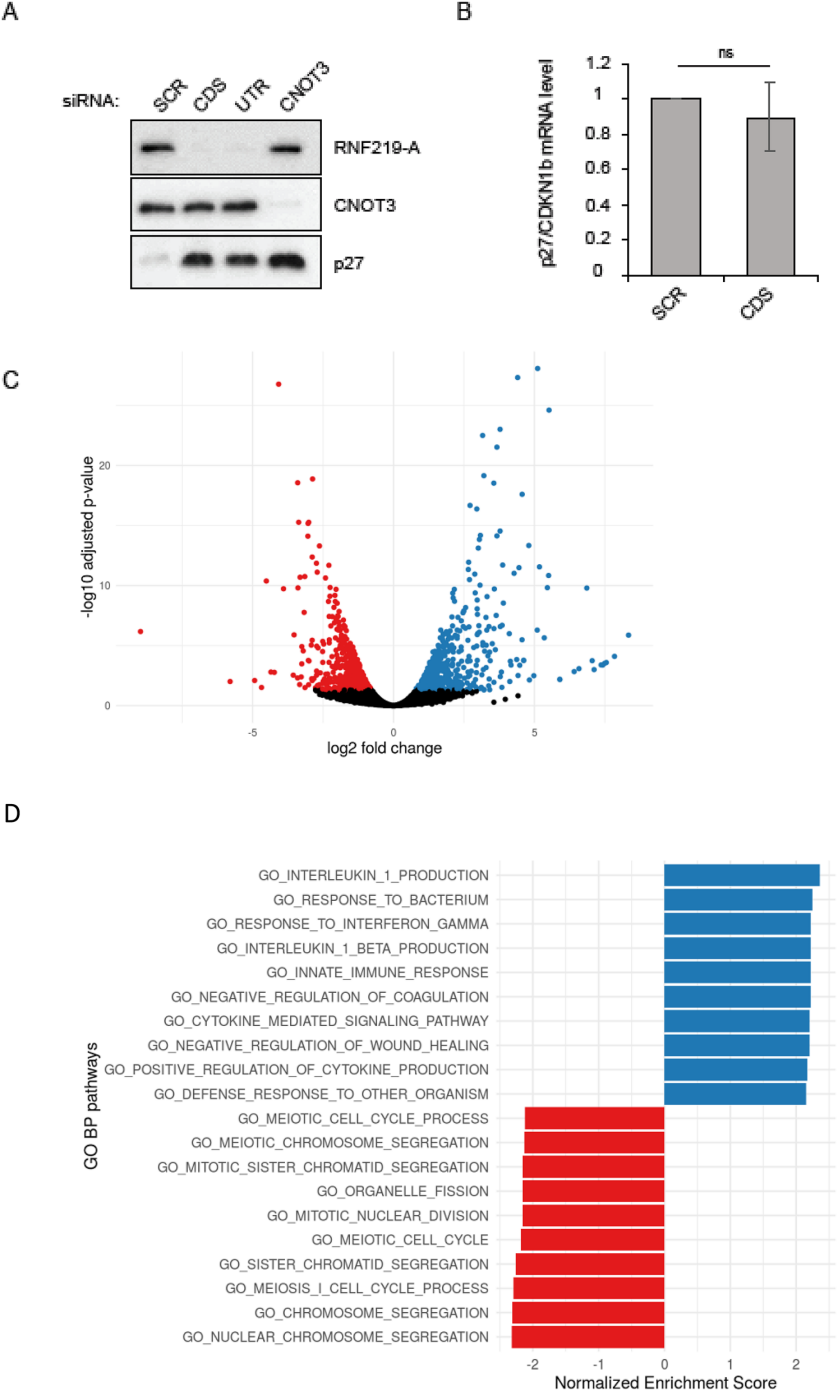
Endogenous RNF219 is implicated in cell cycle regulation. (**A**) Knock-down of RNF219 induces an increase in p27 protein level. HeLa cells were transfected with two siRNA against RNF219 (CDS and UTR) or CNOT3. RNF219, CNOT3 and p27 protein levels were analyzed by immunoblotting using RNF219-A, CNOT3 and p27 antibodies respectively. (**B**) Knock-down of RNF219 does not substantially affect p27 mRNA level. RNA was extracted in control (SCR) or in RNF219 depleted cells (CDS). RT-QPCR was performed using primers specific to p27/CDKN1b (Table S1) and was normalized on 18S mRNA level. p27/CDKN1b mRNA level was set to 1 in the control (SCR). Error bars represent SD, n = 3. ns P > 0.05 based on unpaired two-tailed Student’s *t* test. (**C**) Volcano plot showing differentially expressed (DE) genes of two replicates between siRNF219 CDS versus control SCR. Blue dots represent genes upregulated in RNF219 depleted cells, red represent downregulated genes, black signifying non-DE genes. Differential expression threshold is BH corrected p-value < 0.05 and |LogFC| > 0.5. (**D**) The top 10 downregulated (red) and upregulated (blue) GO Biological Process terms using gene set enrichment analysis comparing siRNF219 CDS versus SCR. All enrichments are significant with a BH corrected p-value < 0.001.

To explore the in vivo function of RNF219, we asked whether RNF219 affects the expression of p27, a known target of CCR4-NOT and member of the Cip/Kip family of cyclin dependent kinase inhibitor that triggers G1 cell cycle arrest^54^. Depletion of RNF219 using two different siRNA (CDS and UTR), triggers an increase of p27 protein level similar to that observed in CNOT3 depleted cells (Fig. 6A). Notably, p27/CDKN1b mRNA levels in RNF219 depleted cells were unchanged compared to control cells (Fig. 6B), suggesting that the effect of RNF219 on p27 is post-transcriptional.

Next, we performed RNA sequencing in RNF219 depleted cells using siRNA targeting the CDS. Differential expression analysis done between control (SCR) and RNF219 depleted cells show that 617 genes were found to be down-regulated while 605 were found upregulated (|logFC| > 0.5 and BH corrected *p*-value < 0.05, Fig. 6C, Table S3). Gene set enrichment analysis of GO terms (Fig. 6D, Table S4) showed that down-regulated genes in RNF219 depleted cells were generally associated with cell cycle regulation and enriched for processes such as chromosome segregation and meiosis. Up-regulated genes were generally associated with innate immune response and enriched for processes such as interleukin 1 production and cytokine signaling.

**Figure 7:**
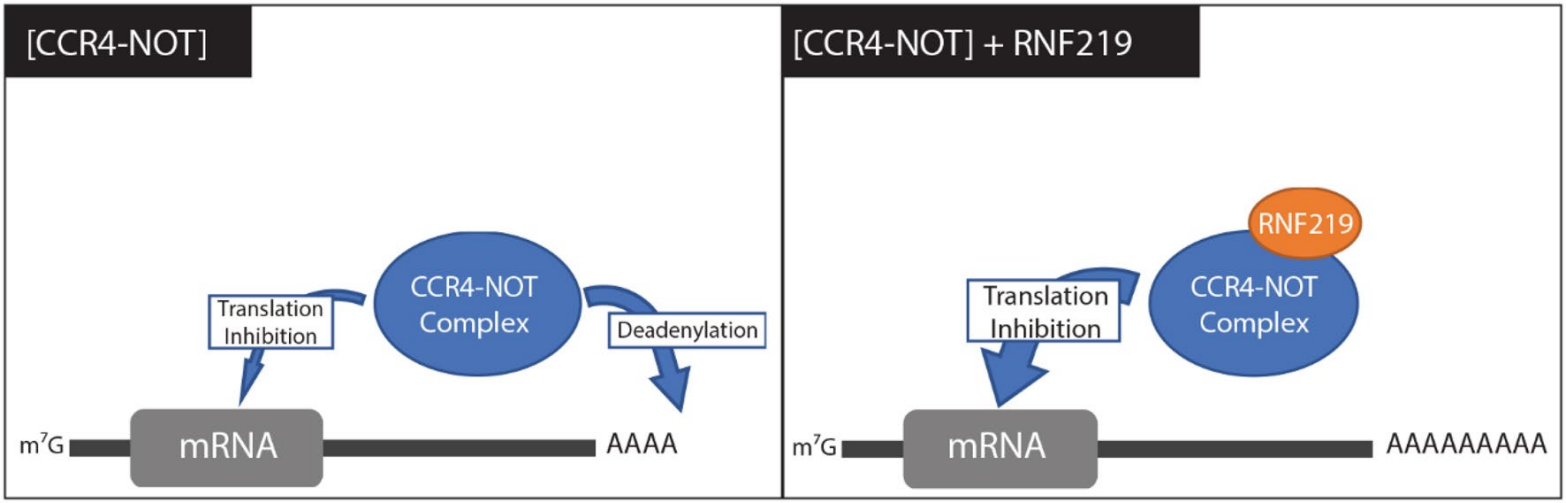
Schematic representation of RNF219 function. Recruitment of RNF219/CCR4-NOT complexes leads to mRNA translational repression in the absence of poly(A) tail deadenylation.

In conclusion, our results altogether suggest that RNF219 is implicated in cell cycle regulation and innate immunity. Moreover, its association with CCR4-NOT represses translation concomitantly to inhibition of deadenylation. We propose that RNF219 could serve as a molecular switch regulating pathway choices between different modes of translation repression (Fig. 7).

## Discussion

In this study we describe RNF219, an emerging E3 ubiquitin ligase, associated with the CCR4-NOT complex. Our biochemical data suggest that RNF219 stably associates with the CCR4-NOT complex (Fig. 2). The results indicate that CNOT1 is required for RNF219 interaction with the NOT module components CNOT2 and CNOT3.

The CCR4-NOT complex is a well-described key regulator of eukaryotic gene expression^55^. Because numerous CCR4-NOT interacting partners regulate the activity of the complex and affect gene expression, RNF219 association with CCR4-NOT (Fig. 2) is an indication that it may also participate in these processes. Consistent with this hypothesis, we find that RNF219 inhibits targeted mRNA expression (Fig. 3) in a manner that greatly depends on its interaction with the CCR4-NOT complex. An exclusively cytoplasmic version of RNF219 is also able to repress targeted mRNA although to a lesser extent compared to WT RNF219 (Fig. 3E). This suggests that RNF219 has a yet unknown nuclear function in regulating mRNA expression. It is likely that it contributes to already described nuclear CCR4-NOT activities such as transcription initiation, elongation, termination, splicing or export to the cytoplasm^55^.

Strikingly, we found that RNF219 affects poly(A) tail shortening. This effect depends on RNF219 interaction with the CCR4-NOT complex (Fig. 5). Knowing that the predominantly described enzymatic activity of the CCR4-NOT complex is mRNA tail deadenylation, it is tempting to suggest that RNF219 acts as a negative regulator of the deadenylase activity of the complex (Fig. 7). During the course of our study, two laboratories provided independent evidence that RNF219 associates with the human CCR4-NOT complex and negatively regulates deadenylation^32,33^.

Furthermore, studies led by Meijer et al. report that eIF4A2 and DDX6, two helicases, compete to interact with CNOT1^56^. While DDX6 stimulates CNOT7 deadenylase activity, eIF4A2 is inhibitory in vitro. Interestingly longer poly(A) tails are bound to eIF4A2, similarly to poly(A) tails observed on the RNF219 targeted reporter^56^. This data supports our model proposing specific interacting partners are able to switch CCR4-NOT function between different modes of translation repression, dependent or independent of deadenylation.

Additionally, two studies reported that post-translational modifications of CCR4-NOT subunits affected its deadenylation activity^52,57^. The first study, Cano et al. identified an E3 ligase, MEX-3C, that interacts with the CCR4-NOT complex and promotes its deadenylase activity by ubiquitylation of the CNOT7 deadenylase^52^. In the second study, Sharma et al. showed that deacetylation of CNOT7 by HDAC1 and 2 stimulates the deadenylase activity of the complex^57^. Thus, post-translational modifications of the CCR4-NOT complex, including acetylation and ubiquitylation, modulate its activity. In contrast to previous reports, RNF219 is the first CCR4-NOT associated E3 ligase potentially inhibiting its enzymatic function. However, in our experiment, RNF219 ubiquitin ligase activity does not appear to be necessary for RNF219 mediated repression in the reporter assay. It is possible that the ubiquitin ligase activity acts at steps, such as RNF219 recruitment or residence time, absent in our tethering assay.

We showed that targeting RNF219 to a reporter mRNA on one hand inhibits its expression (Fig. 3) but on the other hand increased its poly(A) tail length (Fig. 5). This might seem surprising, as longer poly(A) tails have been classically associated with mRNA stabilization and increased translation. We interpret these observations to indicate that the mRNAs that have not been degraded by CCR4-NOT recruitment, and are therefore present in the samples analyzed in the ePat assay, have longer tails due to a protective effect of RNF219. In addition, studies by Lima et al. demonstrated that in somatic cells highly expressed mRNAs tend to have short polyA tails^10^. Consistently, our study reinforces the idea that the relationship between poly(A) tail length and translation efficiency is complex and highly regulated in somatic cells^1,9^.

In conclusion, we show that RNF219 is a novel factor implicated in post-transcriptional regulation of mRNA. Although, direct RNF219 mRNA targets are yet to be discovered, we found that RNF219 affects p27/CDKN1b expression post-transcriptionally, involving RNF219 in cell cycle regulation (Fig. 6). This is consistent with RNF219 function in replication origin firing control and cellular transformation^44^.

To our knowledge RNF219 and eIF4A2 are the first identified modulators of CCR4-NOT activity preventing deadenylation and repressing translation at the same time^56^. Interestingly, Youn et al. have previously described RNF219 and CCR4-NOT interaction using an in vivo proximity-dependent biotinylation (BioID) analysis^58^. This report, defines the core components of stress granules (SGs) and P-Bodies (PBs). It is tempting to speculate that RNF219 modulates CCR4-NOT mediated deadenylation and translational repression of SG or PB associated RNA to allow a rapid cellular response, enabling cells to start protein synthesis from already accumulated mRNAs. Future work will reveal physiologically relevant, direct targets of the RNF219/CCR4-NOT complex and further characterize the pathway of translational repression it regulates.

## Supplementary Information

Supplementary Information 1.

Supplementary Information 2.

Supplementary Information 3.

Supplementary Information 4.

Supplementary Information 5.

Supplementary Legends.

## Data Availability

RNA-sequencing data have been deposited in GEO under the accession number GSE95442. All raw data used for mRNA quantification and luciferase activities are available in Table S5, along with all associated statistical analyses.
