# Supplementary material for: RNF219 regulates CCR4-NOT function in mRNA translation and deadenylation
Source: Sci Rep. 2022 Jun 3;12:9288. doi: 10.1038/s41598-022-13309-8 (PMC9166816; doi:10.1038/s41598-022-13309-8)

Supplemental figure 1

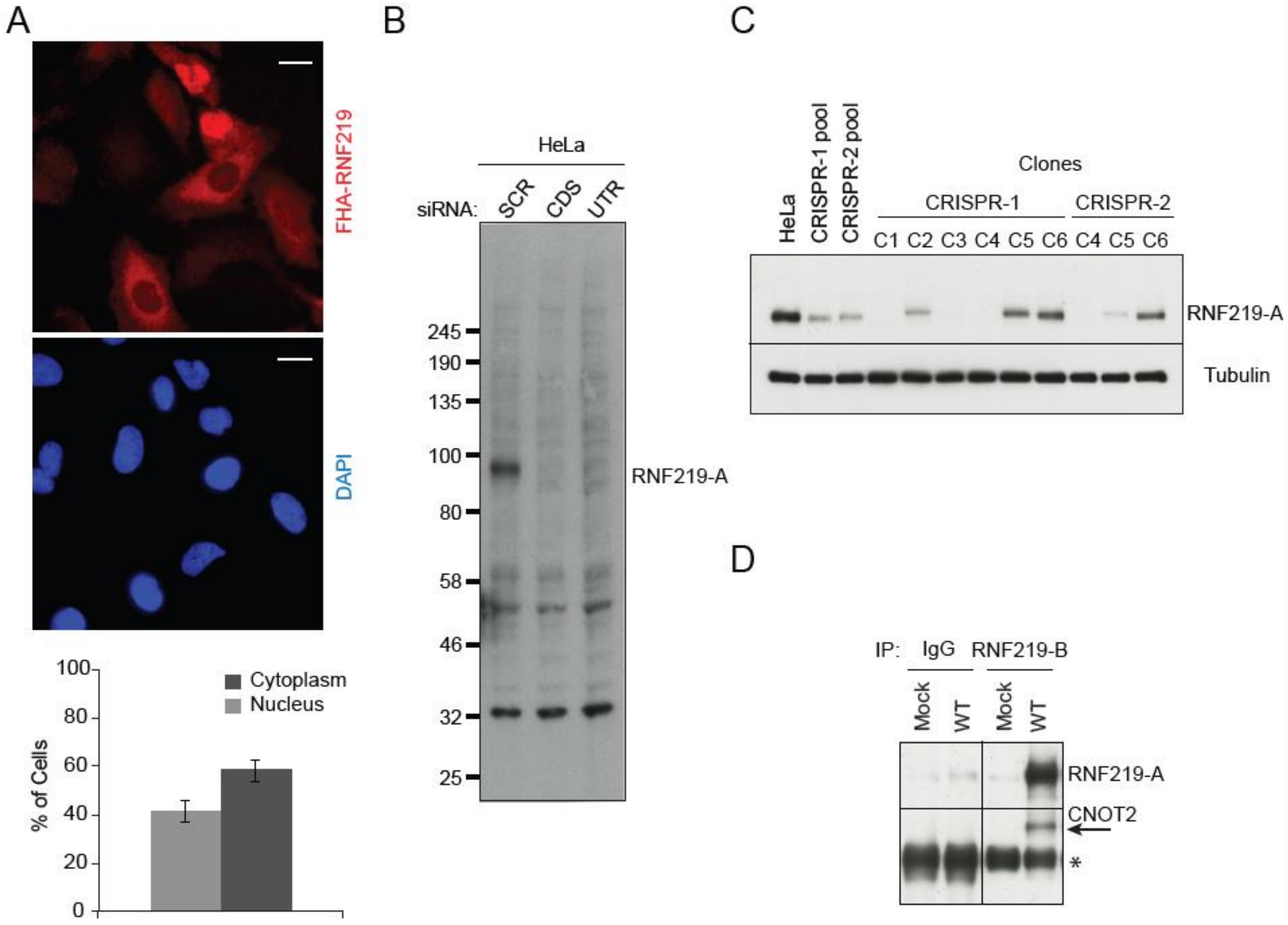

Supplemental figure 2

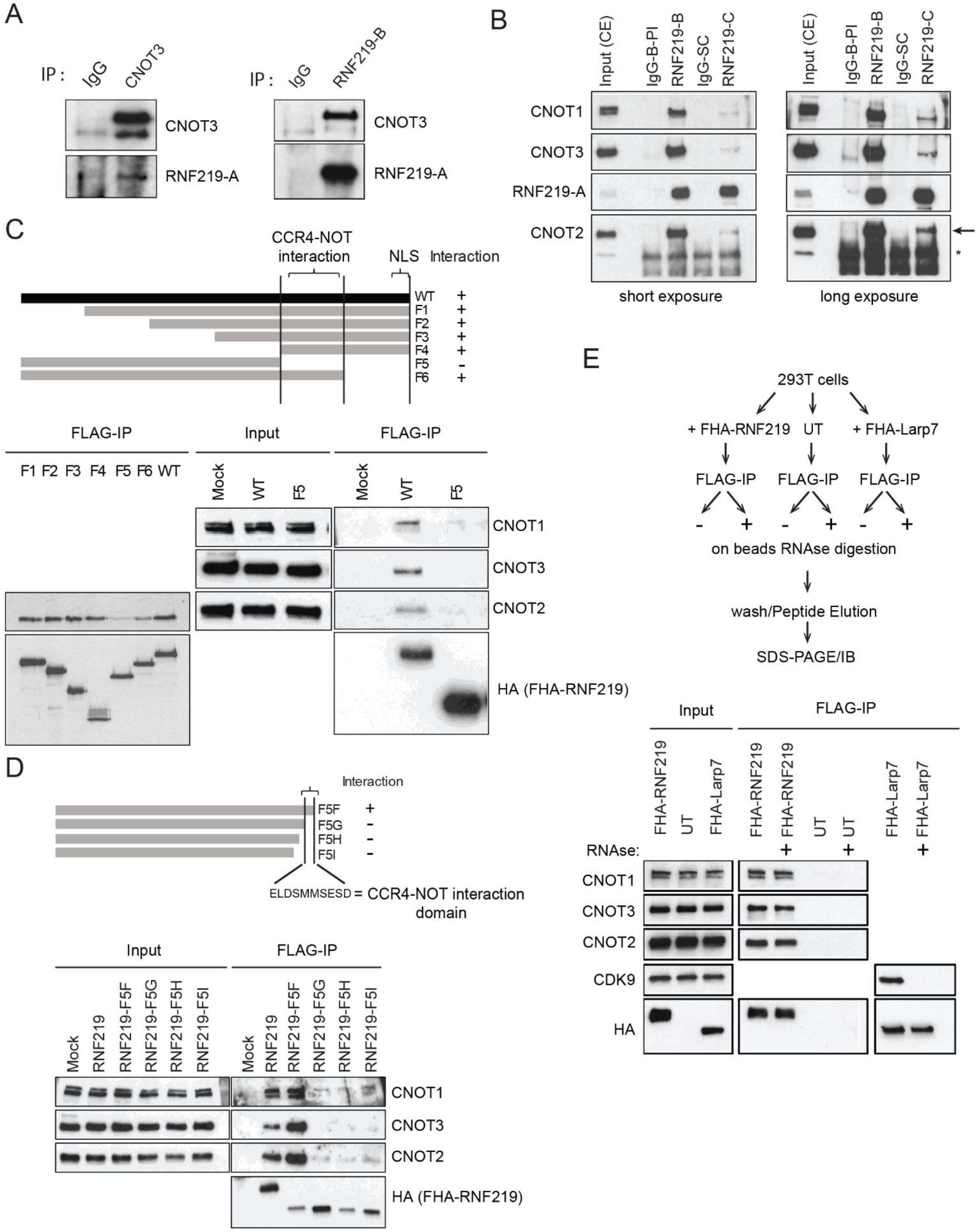

Supplemental figure 3

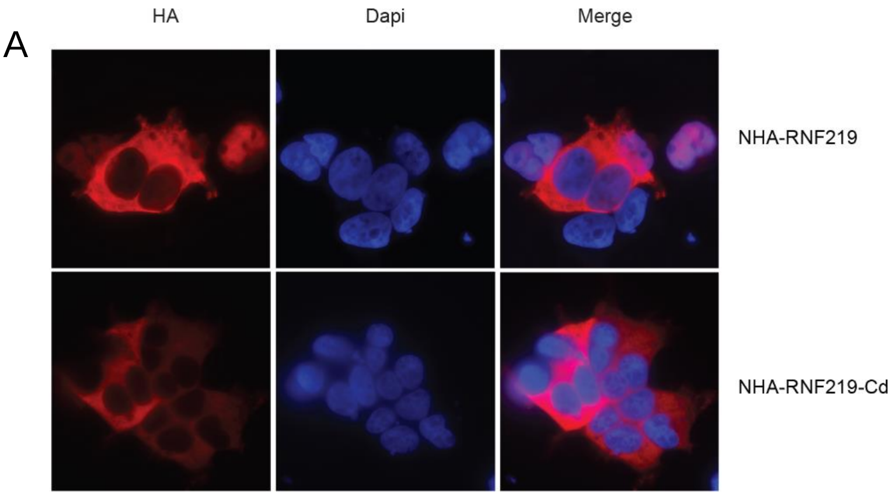

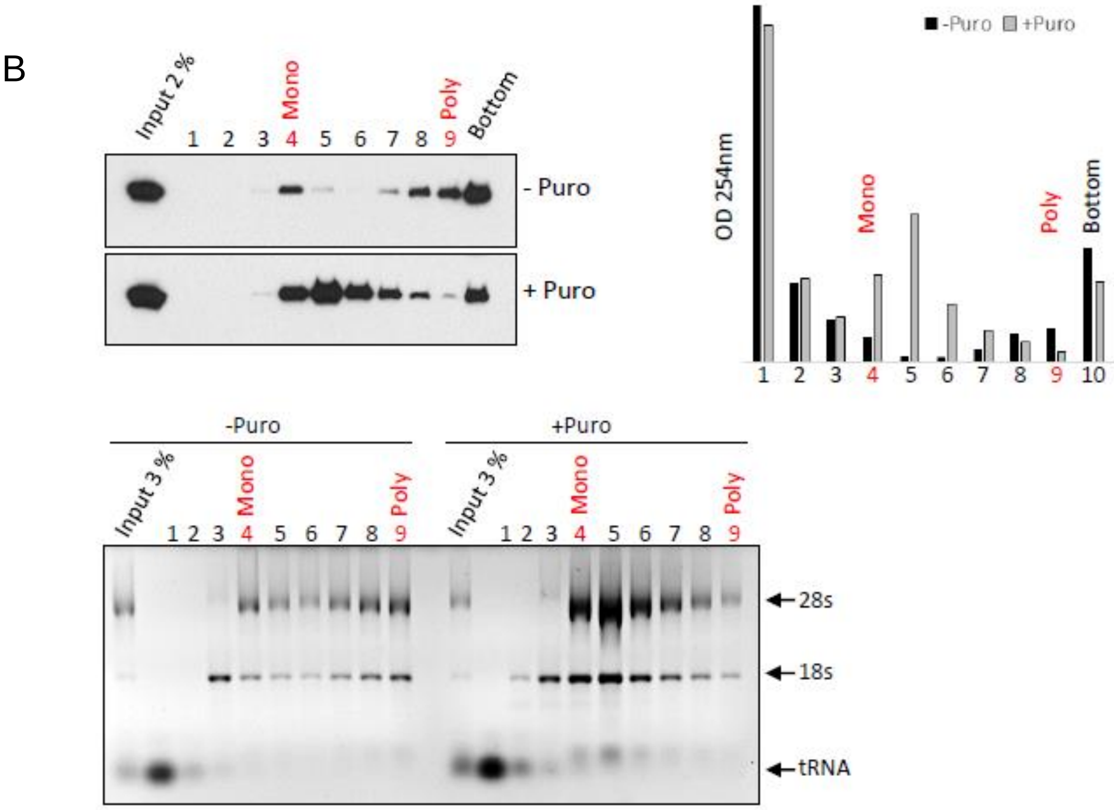

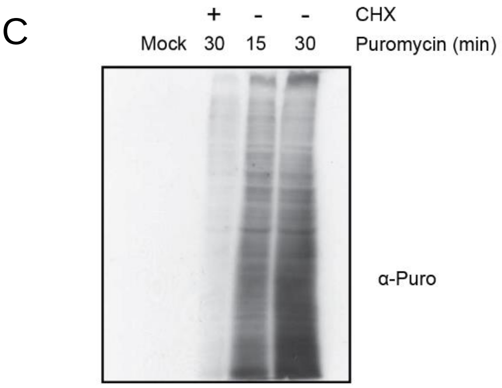

Supplemental figure 4

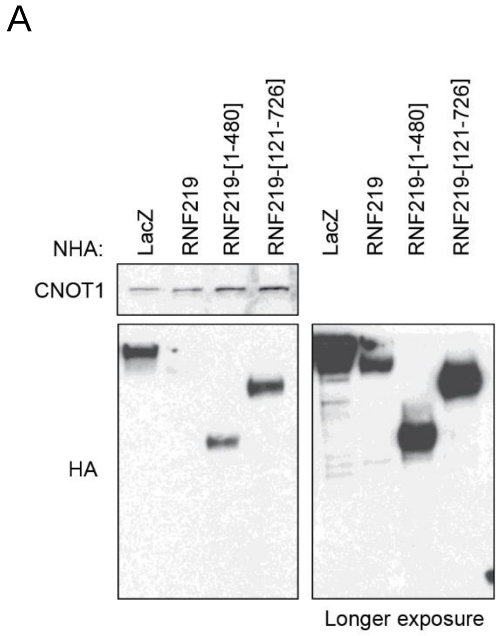

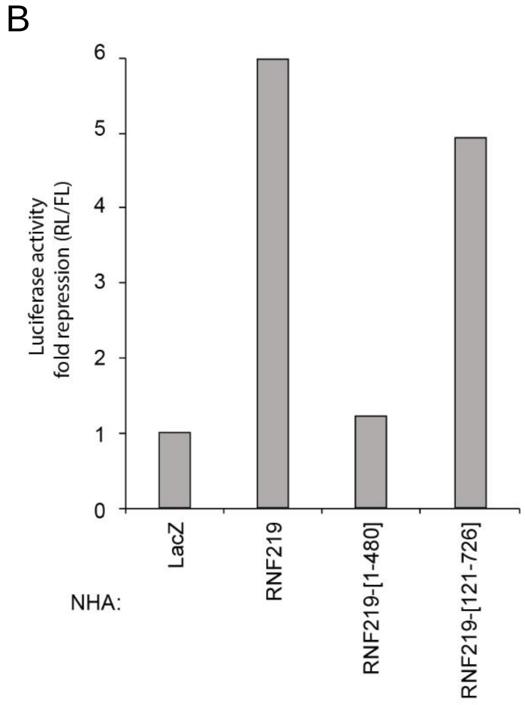

Supplemental Table 1

RTqPCR	RNF219 F	CATCACGTGCCACATTTGCT
	RNF219 R	ATGCTTCCTGACCGTATGGC
	GAPDH F	ACAGTCCATGCCATCACTGCC
	GAPDH R	GCCTGCTTCACCACCTTCTTG
	RENILLA F	TCGTCCATGCTGAGAGTGTC
	RENILLA R	CTAACCTCGCCCTTCTCCTT
	FIREFLY F	TCAAAGAGGCGAACTGTGTG
	FIREFLY R	TTTTCCGTCATCGTCTTTCC

SiRNA	SCRAMBLE	UCUGCAAGGUUAGGCGUCU
	CNOT1_A	GGAACUUGUUUGAAGAAUA
	CNOT1_B	CAAGGUCCUUGGUAUAGUA
	RNF219 CDS	GCAGACCUUAACUGUUCUA
	RNF219 UTR	CUUGUCUUCAGAACUUGGA

ePAT	LUC_1	GCTTTATTTGTGAAATTTGTGATGC
	LUC_2	CGAGCAGACATGATAAGATACATTG
	GAPDH	GAGCCGCACCTTGTCATGTA

Figure 1C

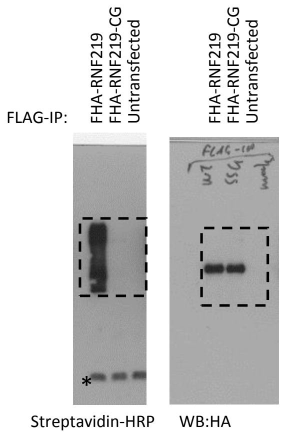

Figure 2B

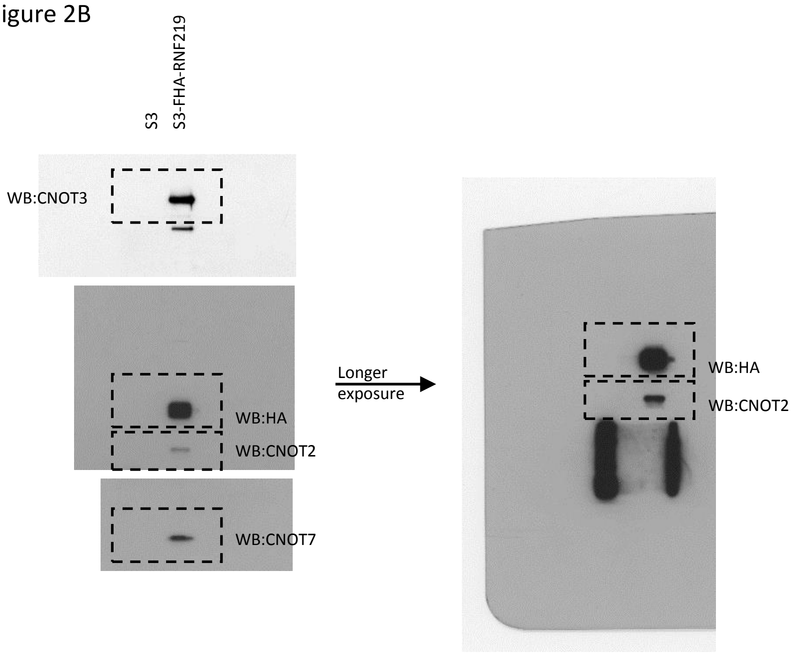

Figure 2C and S2B

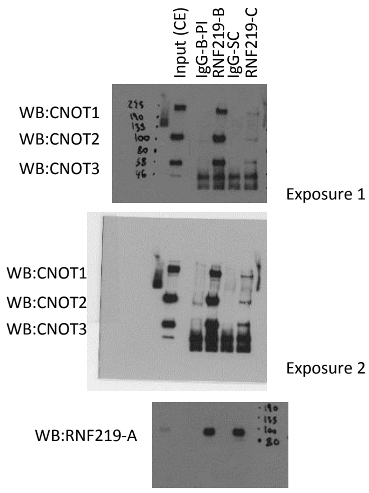

Figure 2D

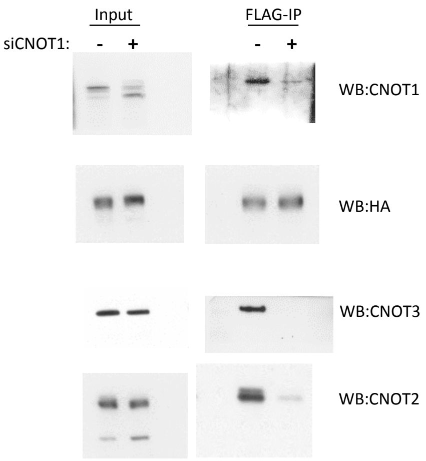

Figure 2F

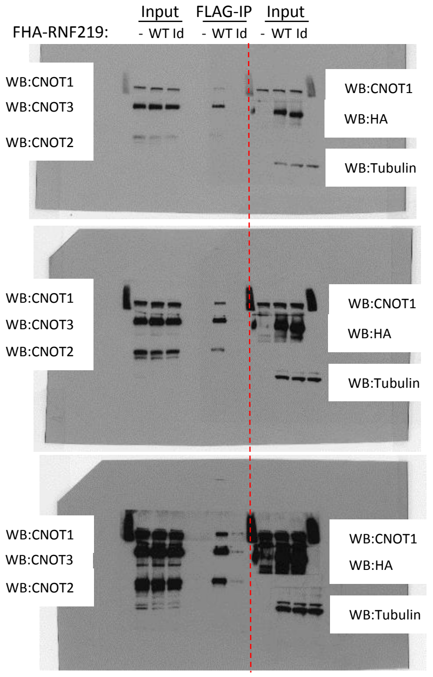

Figure 3B

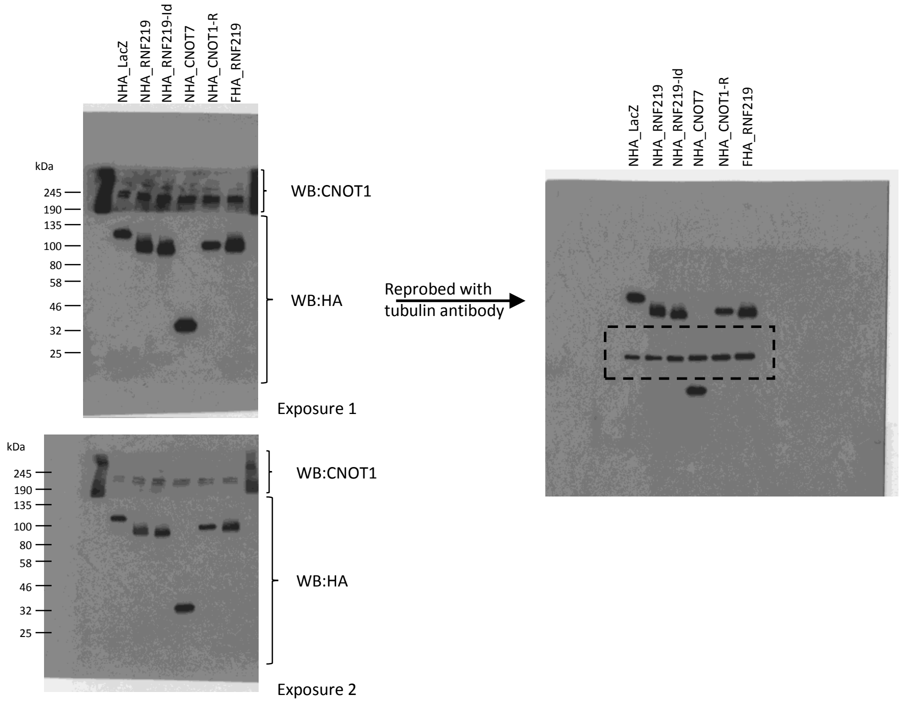

Figure 3G

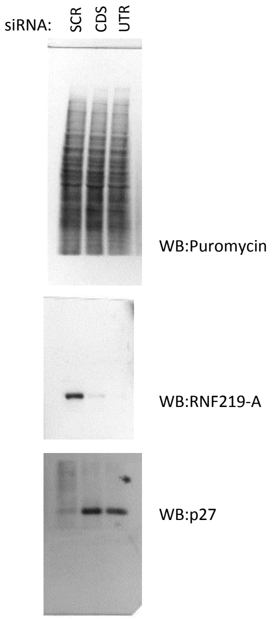

Figure 4A

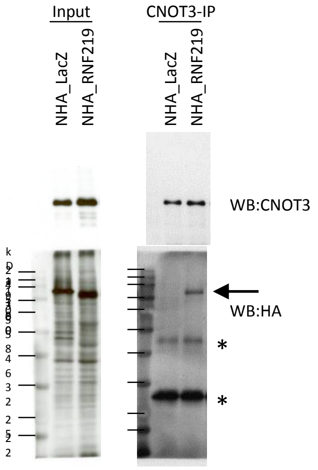

Figure 4D

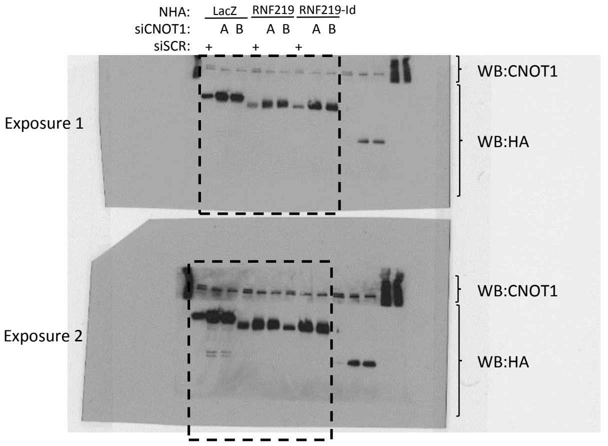

Figure 5A

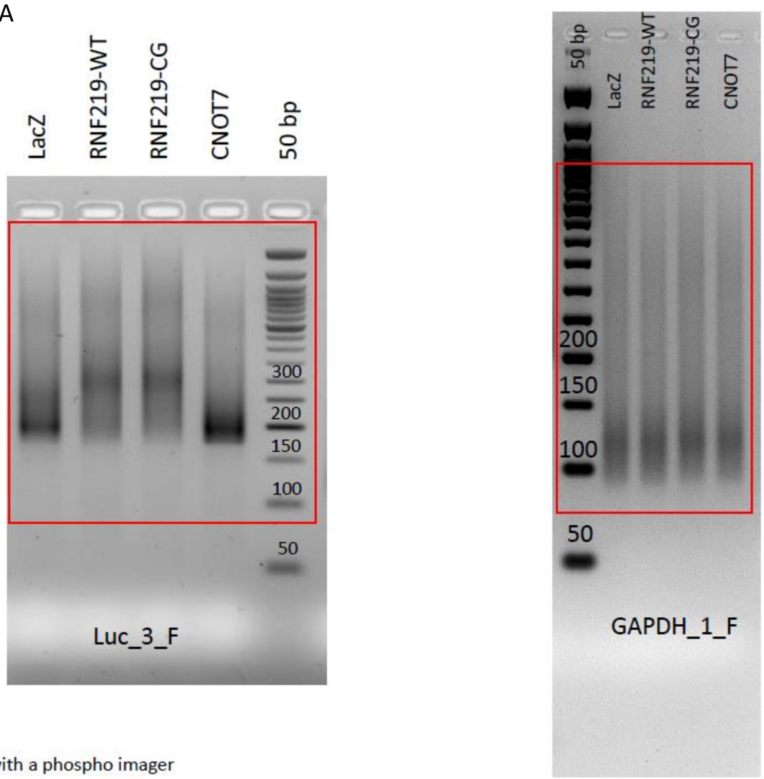

Image taken with a phospho imager

Figure 5B

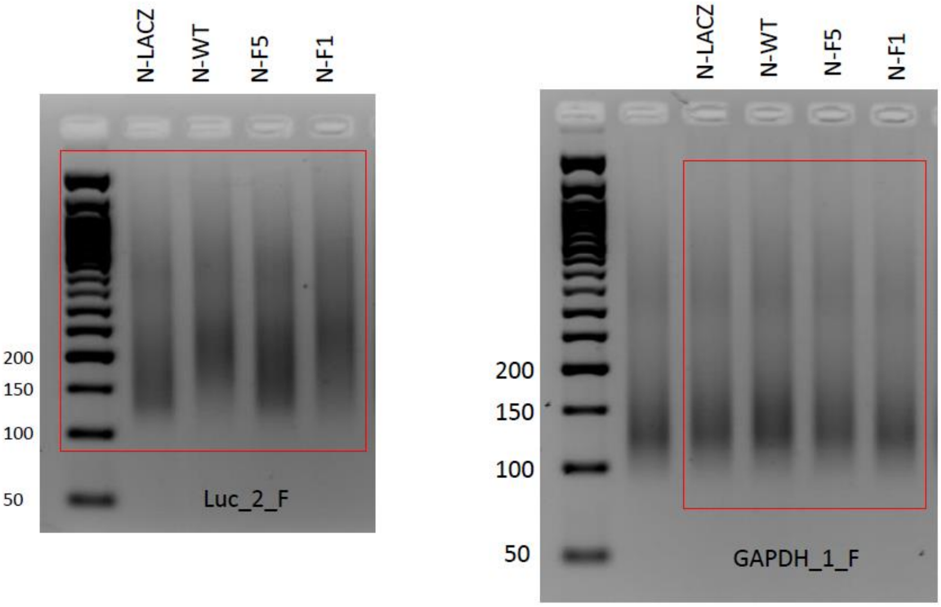

Image taken with a phospho imager

Figure 5C

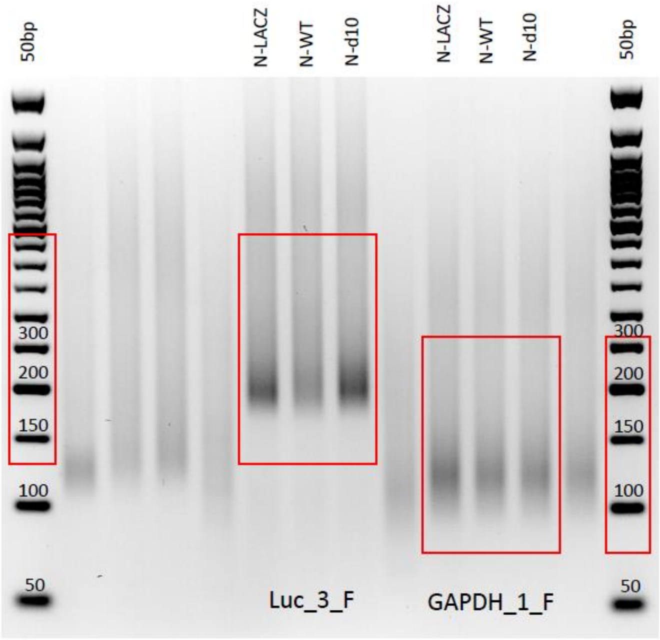

Image taken with a phospho imager

Figure 6A

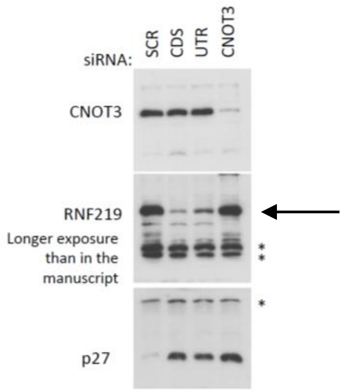

Figure S1C

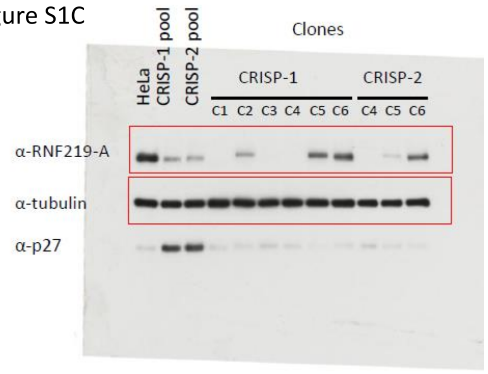

Figure S1D

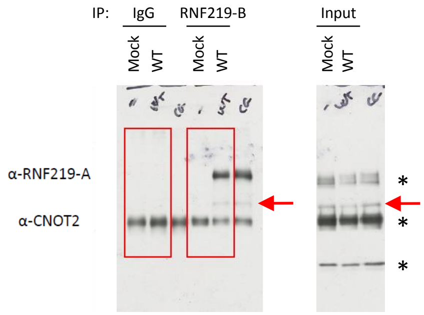

Figure S2A

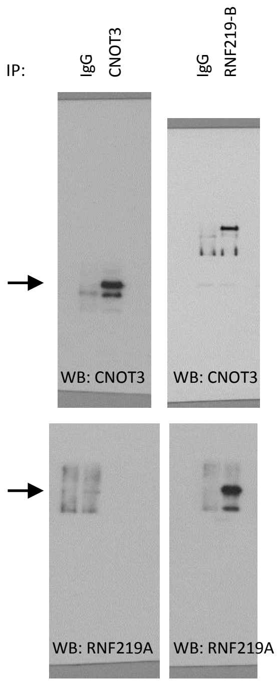

Figure S2C

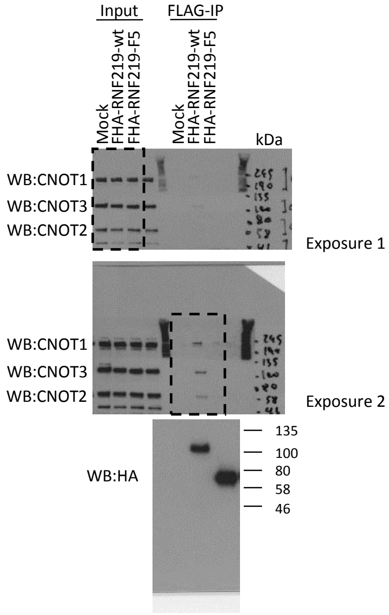

Figure S2C

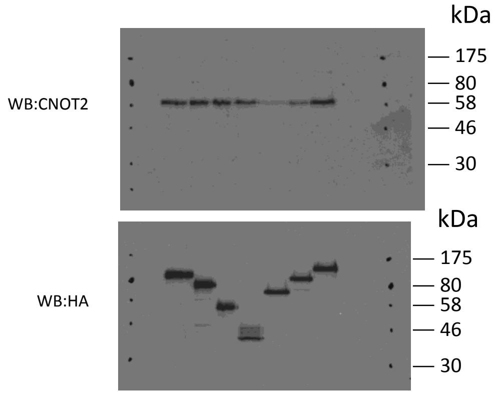

Figure S2D

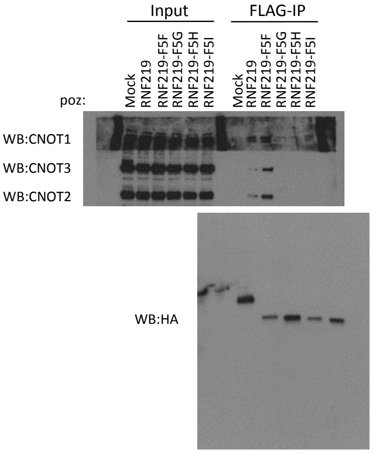

Figure S2E

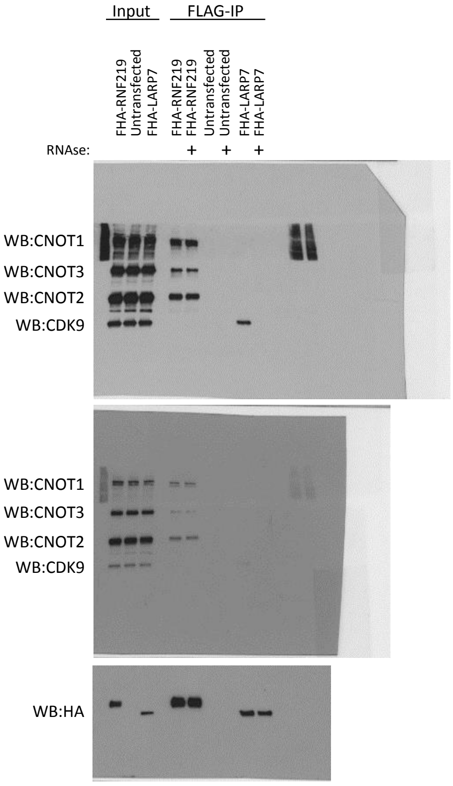

Figure S3

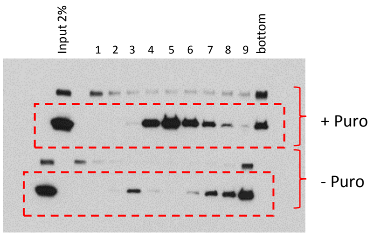

Figure S4

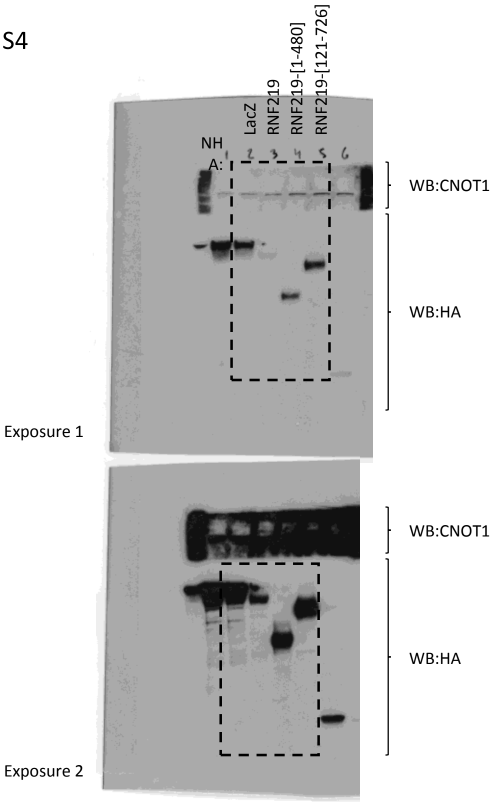

Supplement: Supplementary file 1 — Supplementary Information 1. [file 41598_2022_13309_MOESM1_ESM.pdf]
