# Supplementary material for: RNF219 regulates CCR4-NOT function in mRNA translation and deadenylation
Source: Sci Rep. 2022 Jun 3;12:9288. doi: 10.1038/s41598-022-13309-8 (PMC9166816; doi:10.1038/s41598-022-13309-8)
Supplement: Supplementary file 6 — Supplementary Legends. [file 41598_2022_13309_MOESM6_ESM.docx]

**SUPPLEMENTAL FIGURE LEGENDS**

**Figure S1. RNF219 is a RING dependent ubiquitin ligase**

(A) The subcellular localization of RNF219 is preferentially either nuclear or cytoplasmic. Immuno-fluorescence performed with anti-HA on U2OS cells transfected with the FLAG-HA epitope-tagged RNF219 (FHA-RNF219) construct and nuclei were stained with Dapi (Scale bar, 12 μm). Statistical analysis of immuno-fluorescence experiment above. Error bars represent SD, n = 100.

(B) Immunoblot of extracts from HeLa cells transfected with control siRNA (SCR) or siRNA targeting the coding sequence (CDS) or the 3’UTR (UTR) of the RNF219 mRNA and probed with antibody against endogenous RNF219-A. The control condition (SCR) shows a band at 85 KDa, which is specifically depleted upon CDS and UTR siRNA transfection.

(C) Immunoblot of cell extracts from HeLa cells transfected with a CrispR/Cas9 plasmid expressing Cas9 and 2 selected guide RNA against RNF219 (CRISPR-1 and CRISPR-2). The pools represent mixed populations of transfected cells before clonal selection and Clones represent tested clones from single colony isolations derived from single cells.

(D) RNF219 was immuno-precipitated (IP) from HEK293T CRISPR-1 cells transfected with either pcDNA, a mock plasmid (mock) or FLAG-tagged RNF219 (RNF219) using the homemade RNF219-B antibody. The first 2 lanes contain the purification performed with an irrelevant IgG antibody. The presence of RNF219 in the IP was analyzed by immunoblotting using the RNF219-A and CNOT2 antibodies. The arrow indicates the band corresponding to CNOT2 while the asterisk indicates a non-specific band.

**Figure S2: RNF219 binds the CCR4-NOT complex**

(A) Endogenous CNOT3 (left), endogenous RNF219 (middle) or endogenous CNOT1 (right) was immuno-purified from HeLa cell extracts using an anti-CNOT3, anti-RNF219 (RNF219-B), anti-CNOT1 or irrelevant IgG antibody. IP were analysed by IB with indicated antibodies.

(B) RNF219 was immunoprecipitated (IP) using two different RNF219 specific antibodies (RNF219-B and RNF219-C) from HEK293T cell extracts. IP were analysed by IB with indicated antibodies. CCR4-NOT subunits were specifically enriched with both antibodies used for IP. IgG-B-PI (Pre-immune IgG antibody) and IgG-SC (a commercial control antibody) were used as control antibodies. The arrow indicates the band corresponding to CNOT2 while the asterisk indicates a non-specific band.

(C) RNF219 protein was truncated in multiple fragments until loss of CCR4-NOT interaction. RNF219 was truncated in six large fragments with a truncation step of 121 amino acids (represented by F1 to F6). The Flag-HA tagged fragments were transfected in HEK293T cells and purified with M2-FLAG beads. Peptide eluted material was analysed by IB with indicated antibodies. The F5 truncation lost interaction with three CCR4-NOT subunits (CNOT1, CNOT2, CNOT3). The Mock sample corresponds to cells transfected with a mock plasmid: pcDNA.

(D) The F5 truncation, which lost interaction with 3 CCR4-NOT subunits (CNOT1, CNOT2, CNOT3), was truncated in four fragments with a truncation step of 10 amino acids (represented by F5F, F5G, F5H, F5I). The Flag-HA tagged fragments were transfected in cells. The Mock sample corresponds to cells transfected with a mock plasmid: pcDNA. IP were performed with M2-FLAG beads on cell extracts and analysed by IB with indicated antibodies.

(E) The interaction between RNF219 and the CCR4-NOT complex is RNase resistant. Top: the experimental scheme is shown. Bottom: FLAG and HA tagged RNF219 (FHA-RNF219) or FHA-Larp7 were IP’d on FLAG beads which were subjected to mock or RNase treatment. Co-IP’d proteins were analysed by IB. The RNase sensitive Larp7/CDK9 interaction served as positive control for RNase digestion.

**Figure S3.** **RNF219 affects the translation of a targeted mRNA**

(A) Immuno-localization of NHA-RNF219 and NHA-RNF219-Cd. Immuno-fluorescence was performed with anti-HA antibody on HEK293T cells transfected with the indicated constructs and showed exclusively cytoplasmic staining for NHA-RNF219-Cd protein.

(B) (Top, left) Immunoblot of sucrose gradient fractions with RPL7a antibody. Fractions 4 and 9 were processed to measure the reporter mRNA level in the monosomal (Mono) and polysomal fraction (Poly) respectively in Fig. 3F. (Top, right) OD254 of sucrose gradient fractions. (Bottom) Ethidium bromide stained agarose gel of total RNA from sucrose gradient fractions.

(C) Translation efficiency of HeLa cells is monitored by quantifying Puromycin incorporation in nascent protein. Immunoblot using anti-puromycin antibody is performed on total cell extract after puromycin pulse ^50^. In the mock condition, no puromycin is added to the medium. In the second lane, the translation inhibitor Cycloheximide is added at the same time for 30 min. In the third and fourth lanes, puromycin pulses were of 15 and 30-min respectively.

**Figure S4. RNF219 affects the polyA tail length of a targeted mRNA**

(A) Immunoblot of experiment shown in Figure 5B.

(B) Renilla luciferase activity normalized to Firefly luciferase activity of experiment shown in Figure 5B.

**Supplementary Table S1:**

List of primers used in this study

**Supplementary Table S2:**

(Top) List of RNF219 interacting proteins detected by MS/MS with high score. CCR4-NOT subunits are highlighted in yellow and further with red in sample with highest coverage Known CCR4-NOT recruiting proteins are highlighted in blue. (Bottom) Colloidal blue stained gel used for MS/MS analysis. Sample numbers corresponding to the table, combining bands or regions of the gel, are in red.

**Supplementary Table S3:**

Results of the differential expression analysis between control (SCR) and RNF219 depleted

cells (CDS). The differential expression analysis was done using DESeq2. A negative Log2FC value corresponds to genes downregulated in RNF219 depleted cells with respect to the control, while a positive Log2FC indicates genes upregulated in RNF219 depleted cells. Padj corresponds to the BH correct p-values.

**Supplementary Table S4:**

Results of the gene set enrichment analysis between control (SCR) and RNF219 depleted cells (CDS). GSEA was done using the R package fgsea and the GO biological process pathway list (c5.go.bp.v7.2.symbols.gmt) from the GSEA/MSigDB website (https://www.gsea-msigdb.org/).

**Supplementary Table S5:**

Luciferase measurements, qPCR results and associated statistical analysis.
